# Folate levels modulate oncogene-induced replication stress and tumorigenicity

**DOI:** 10.15252/emmm.201404824

**Published:** 2015-07-21

**Authors:** Noa Lamm, Karin Maoz, Assaf C Bester, Michael M Im, Donna S Shewach, Rotem Karni, Batsheva Kerem

**Affiliations:** 1 Department of Genetics, The Alexander Silberman Institute of Life Sciences, Edmond J. Safra Campus, The Hebrew University of JerusalemJerusalem, Israel; 2 Department of Pharmacology, University of Michigan Medical CenterAnn Arbor, MI, USA; 3 Department of Biochemistry and Molecular Biology, Institute for Medical Research Israel-Canada, The Hebrew University-Hadassah Medical SchoolJerusalem, Israel

**Keywords:** cancer development, chromosomal instability, folate deficiency, oncogene expression, replication stress

## Abstract

Chromosomal instability in early cancer stages is caused by replication stress. One mechanism by which oncogene expression induces replication stress is to drive cell proliferation with insufficient nucleotide levels. Cancer development is driven by alterations in both genetic and environmental factors. Here, we investigated whether replication stress can be modulated by both genetic and non-genetic factors and whether the extent of replication stress affects the probability of neoplastic transformation. To do so, we studied the effect of folate, a micronutrient that is essential for nucleotide biosynthesis, on oncogene-induced tumorigenicity. We show that folate deficiency by itself leads to replication stress in a concentration-dependent manner. Folate deficiency significantly enhances oncogene-induced replication stress, leading to increased DNA damage and tumorigenicity *in vitro*. Importantly, oncogene-expressing cells, when grown under folate deficiency, exhibit a significantly increased frequency of tumor development in mice. These findings suggest that replication stress is a quantitative trait affected by both genetic and non-genetic factors and that the extent of replication stress plays an important role in cancer development.

## Introduction

Chromosomal instability is a hallmark of nearly all solid tumors and adult-onset leukemias (Hanahan & Weinberg, 2011). Enormous efforts have been made in the last few decades to understand the cellular and environmental factors leading to genomic instability and cancer development (Lengauer *et al*, 1998; McGranahan *et al*, 2012; Ozeri-Galai *et al*, 2012). In recent years, it has become apparent that in early stages of cancer development, DNA instability is caused by perturbed DNA replication (Ames & Wakimoto, 2002; Gorgoulis *et al*, 2005; Tsantoulis *et al*, 2008). This replication stress is defined as perturbations in the dynamics of the replication machinery and is characterized by slow fork progression, and in some cases even fork collapse, activation of additional origins, and asymmetric progression of replication forks emerging from the same origin (Hills & Diffley, 2014). In the early stages of cancer development, oncogene activation leads to replication stress (Bartkova *et al*, 2005; Di Micco *et al*, 2006; Tsantoulis *et al*, 2008; Bester *et al*, 2011), which underscores the role of DNA replication in cancer development (Halazonetis *et al*, 2008; Negrini *et al*, 2010). Several mechanisms by which oncogenes induce replication stress were recently identified, including insufficient nucleotide pools to support the extensive enforced DNA replication (Bester *et al*, 2011; Mannava *et al*, 2013), interference with the pre-replication complex assembly (Ekholm-Reed *et al*, 2004) and the collision between replication and transcription (Jones *et al*, 2013). However, it remains unclear whether the extent of the replication stress can affect the probability of neoplastic transformation. Moreover, whether enhanced replication stress can be driven by a combination of genetic, cellular, and environmental factors is largely unknown.

Micronutrients are important environmental factors for normal cellular proliferation. Suboptimal levels (a deficiency) of micronutrients increase the risk of many types of cancer (reviewed in (Vidal *et al*, 2011; Ames & Wakimoto, 2002). One classic example of such a micronutrient is folate, a B9 water-soluble vitamin found mainly in green leafy vegetables (Camilo *et al*, 1996). Folate is the general term for many derivatives found in intracellular equilibrium, which except for *de novo* synthesis by intestinal microflora cannot be produced by most mammals (Camilo *et al*, 1996). Folic acid is the fully oxidized monoglutamyl form of folate, which is frequently used as a nutritional supplement. Therefore, folate must be obtained from dietary or supplementary sources (Shane, 1989). Folate is required for one-carbon transfer reactions including the synthesis of thymidine and purines and the methylation of cytosines in DNA (reviewed in (Duthie, 2011; Kim, 1999b; Shane, 1989). It has been shown that folate deficiency caused by the use of antifolate reagents perturbs the size and balance of the nucleotide pool (Shane, 1989). However, the effect of folate deficiency on DNA replication dynamics remains unclear.

Many epidemiological studies have shown that suboptimal levels of folate are associated with several types of cancer, including colon (Giovannucci *et al*, 1995; Zhang *et al*, 1999; Rohan *et al*, 2000), cervical (Rampersaud *et al*, 2002; García-Closas *et al*, 2005), gastric, and esophageal cancers (Mayne *et al*, 2001). Studies in human cultured cells and *in vivo* studies in both animal models and humans have shown that severe folate deficiency is associated with double-strand breaks (DSBs), chromosome instability, and micronuclei formation (Chen *et al*, 1989; James & Yin, 1989; Duthie & McMillan, 1997; MacGregor *et al*, 1997; Pogribny *et al*, 1997; Melnyk *et al*, 1999; Duthie *et al*, 2000a,b, 2008; Beetstra *et al*, 2005). The main mechanism linking folate deficiency to DNA damage is presumed to be the incorporation of dUMP into the DNA, which is thought to culminate in futile cycles of uracil excision, single-strand breaks, and possibly chromosomal breakage (Blount *et al*, 1997). Importantly, it was shown that folate deficiency enhances the activity of various chemical carcinogens in numerous organs (Eto & Krumdieck, 1986). To date, however, a mechanism that can account for the co-carcinogenic role of folate deficiency has yet to be found.

Folate deficiency has a dual effect on the tumorigenic potential of the cells depending on the duration and extent of the folate deficiency and on the cell stage (tumorigenicity). In neoplastic cells, there is extensive DNA replication and cell division. In these cells, folate deficiency causes ineffective DNA synthesis, resulting in inhibition of tumor growth (Kim, 1999a,b; Choi & Mason, 2002). Indeed, this has been the basis for cancer chemotherapy using a number of antifolate agents (e.g., methotrexate and 5-fluorouracil) (Kim, 1999a,b; Choi & Mason, 2002). Like most chemotherapies, antifolate drugs are toxic to both normal and neoplastic cells and prolonged folate deficiency eventually results in growth arrest and cell death regardless of the tumorigenicity of the cells. However, under shorter and milder folate deficiency conditions, neoplastic cells and other extensive proliferating cells will die, whereas normal cells will survive. An accumulating body of epidemiological, clinical, and experimental evidence suggests that normal cells that survived folate deficiency are predisposed to neoplastic transformation (Kim, 1999a,b, 2003). This dual effect of folate deficiency, which is also known as the “double-edged sword” effect, explains why methotrexate therapy is associated with increased risk of secondary malignancy (Schmiegelow *et al*, 2009).

In the current study, we investigated the combined effect of genetic and dietary factors on replication dynamics, genome stability, and cancer development. Our results show that suboptimal levels of folate lead to replication stress and DSBs in a concentration-dependent manner. Importantly, folate deficiency significantly enhances oncogene-induced replication stress, DNA damage, and tumorigenicity *in vitro*. Furthermore, oncogene-expressing cells grown under folate deficiency show a significant increase in the frequency of tumor development in mice. These findings suggest that replication stress is a quantitative trait that can be affected by both genetic and non-genetic (e.g., dietary) factors.

## Results

### Folate deficiency perturbs cellular DNA replication dynamics

**Figure 1:**
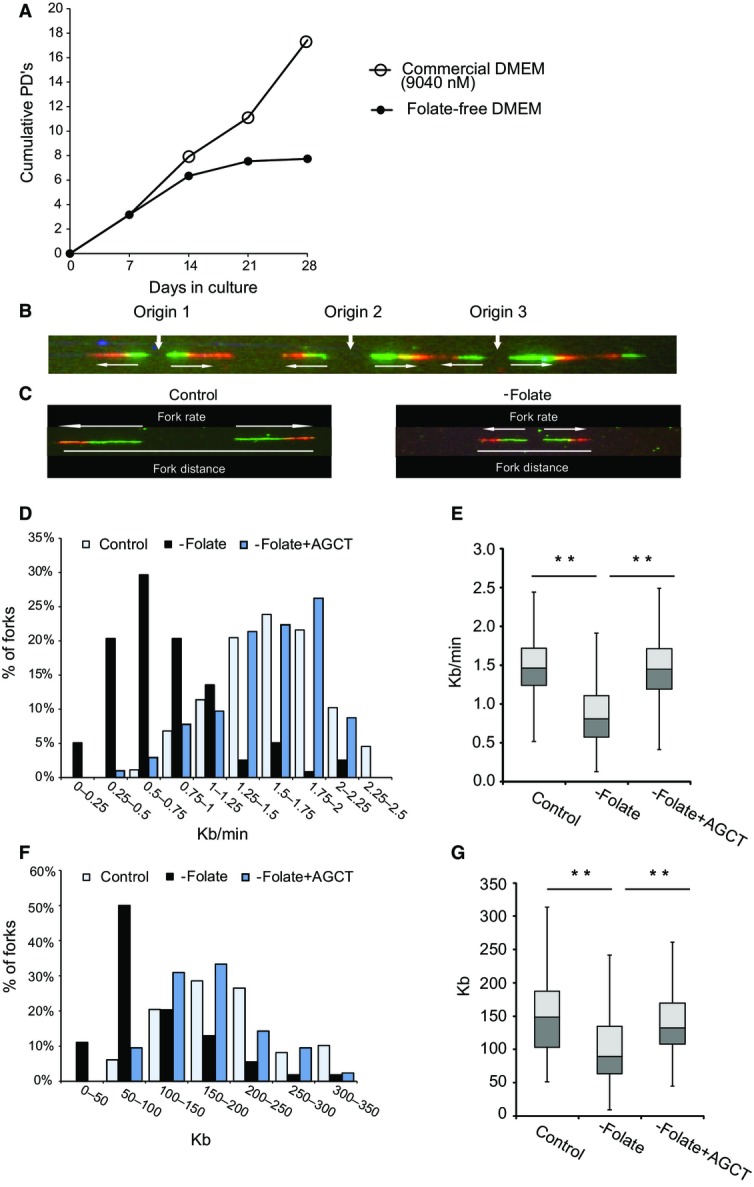
Growth rate and replication dynamics in BJ cells grown in a folate-free medium with and without nucleoside supplementation Population doublings (PDs) determined in BJ cells cultured with and without folate for 28 days.

To investigate the role of folate levels in tumorigenesis, we first analyzed the effects of folate deficiency on DNA replication dynamics. For this purpose, immortalized primary foreskin fibroblasts (BJ-hTert) were grown for 7 days in a folate-free medium (folate-free DMEM). During this time, the folate-deficient cells exhibited a similar growth rate as their counterparts that were cultured in a normal medium (Fig1A), indicating that differences between the cultures were not a result of impaired growth. To investigate the effect of the folate-free medium on cellular DNA replication, we took advantage of the high-resolution DNA combing approach which enables replication analysis on single DNA molecules. The newly synthesized DNA, labeled with IdU and CldU, can be detected by fluorescent antibodies (green and red, respectively) (Fig1B). First, we analyzed the effect of folate deficiency on the cellular replication fork rate (Fig1C and D). The results showed a dramatic decrease in the mean replication rate, from 1.59 Kb/min in cells cultured in a normal medium to 0.78 Kb/min in cells grown in a folate-free medium (*P* < 1.6 × 10^−32^). Importantly, a dramatic increase in the percentage of very slow forks (0.75 Kb/min and below) was observed following growth in a folate-free medium (from 3% under normal conditions to 54% under folate deficiency; Fig1D). Similar results were obtained in three independent experiments (Fig1E; Appendix Fig S1A). These results indicate that folate deficiency leads to a significant decrease in fork progression rate.

Example of a single combed DNA molecule labeled with IdU (green) and CldU (red), showing replication from three adjacent origins. Horizontal white arrows indicate fork orientation.

Representative examples of single combed DNA molecules from control cells and cells grown for 7 days in a folate-free medium.

Fork rate (Kb/min) distribution. Light blue bars: BJ cells (*n *=* *126); black bars: BJ cells that were cultured for 7 days in a folate-free medium (*n *=* *131); blue bars: BJ cells cultured for 7 days in a folate-free medium and supplemented with A, G, C, and T nucleosides for the last 48 h of the experiment (*n *=* *138).

Box plot summarizing the fork rate distribution (Kb/min) of three independent experiments. Control (*n* = 360); −folate (*n *=* *372); −folate + AGCT (*n *=* *361).

Fork distance (Kb) distribution. The color code is as in (D). Control (*n *=* *72); −folate (*n *=* *71); −folate + AGCT (*n *=* *75).

Box plot summarizing the fork distance distribution (Kb) of three independent experiments. Control (*n *=* *212); −folate (*n *=* *215); −folate + AGCT (*n *=* *209). Data information: (E, G) Main box represents the values from the lower to upper quartile (25^th^ to 75^th^ percentile). The middle line represents the median. **P <* *0.0001.

When DNA replication is perturbed, the number of active origins increases in an attempt to compensate for the slow fork progression (Anglana *et al*, 2003; Ge *et al*, 2007; Courbet *et al*, 2008). For this reason, we studied the effect of growth in a folate-free medium on origin density by measuring the distance between two sister forks, which in unsynchronized cells is approximately half of the replicon length (Maya-Mendoza *et al*, 2007). The replicon length scales with increasing inter-origin distances and is therefore a readout of the distance between activated origins. The results showed a significant decrease in the mean fork distance from 195 Kb in the control cells to only 107 Kb in the folate-deficient cells (*P* < 4 × 10^−11^) (Fig1C and F). Similar results were obtained in three independent experiments (Fig1G; Appendix Fig S1B). Altogether, these results indicate that folate deficiency leads to dramatic replication perturbations. We hypothesized that this observed replication stress was due to an insufficient nucleotide pool generated by folate deficiency. For this purpose, BJ cells were grown for 7 days in a folate-free medium and were supplemented with 50 μM of each of the four nucleosides for the last 48 h. Evaluating the replication dynamics using DNA combing revealed that the exogenous supply of nucleosides almost completely restored the average fork rate (Fig1D and E; Appendix Fig S1A) and the average fork distance (Fig1F and G; Appendix Fig S1B). Using the high-performance liquid chromatography (HPLC) method, the concentrations of the cellular dNTPs were measured. As expected, the concentration of the cellular dTTP in cells grown under folate deficiency for 15–30 days was significantly reduced compared to the concentration in same cells grown in a normal medium (Appendix Fig S2). The levels of the dATP, dGTP, and dCTP were below detection. Since the level of dTTP in the folate-deficient medium is very low, uracil misincorporation into the DNA in the cells is expected (Duthie & Hawdon, 1998; Fenech, 2012).

### The extent of replication stress is affected by the levels and duration of folate deficiency

In cultured cells, a folate concentration in the 12–120 nM range was shown to be negatively correlated with DNA damage and micronuclei formation (reviewed in Fenech, 2012). Whereas 20 nM is considered a severe folate deficiency in tissue cultured cells and 100 nM is considered to be mild, 500 nM has not, to the best of our knowledge, been reported to induce any DNA damage. Hence, we studied the effect of different folate concentrations on replication dynamics. We grew BJ cells in a folate-free medium and in a medium containing 20, 100, 500, and 9,040 nM folate. The latter is the regular concentration in the commercial DMEM.

**Figure 2:**
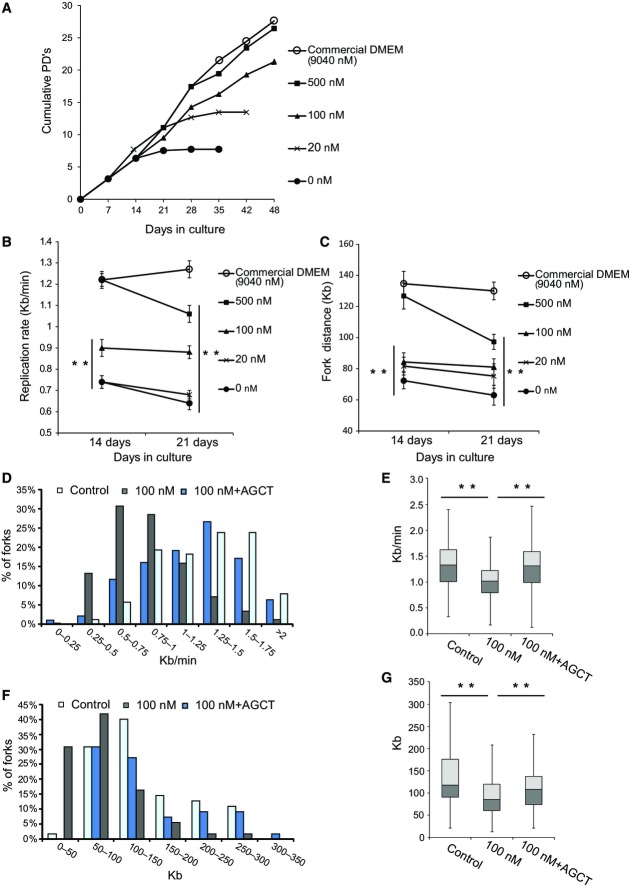
Growth rate and replication dynamics in BJ cells grown under various folate concentrations with and without nucleoside supplementation A Population doublings (PDs) determined in BJ cells cultured at the indicated folate concentrations for 48 days.

First, the effect of various folate concentrations on cell growth was studied by analysis of population doublings (PDs). As can be seen in Fig2A, the effect was concentration dependent. Cells cultured with 500 nM folate showed a similar growth rate as control cells during the 48 days of culturing, whereas cells cultured with 100 nM folate showed a reduced growth rate, but continued to grow during the whole experiment. In contrast, cells cultured with 20 nM folate showed a major decrease in growth rate starting at ∼21 days of culturing and stopped growing after ∼35 days. The effect of the folate-free medium was even stronger, leading to growth arrest after only 21 days (Fig2A).

B, C The average replication rate ± SEM (B) and the average fork distance ± SEM (C) in the indicated folate concentrations at 14 and 21 days. At least 115 DNA fibers were analyzed at each concentration and at each time point to determine the average replication rate. At least 71 replication forks were analyzed at each concentration and at each time point to determine the average fork distance.

D–G BJ cells were grown for 14 days in 100 nM folate with and without nucleoside supplementation. (D) Fork rate (Kb/min) distribution. Light blue bars: BJ cells (*n *=* *115); gray bars: BJ cells that were cultured for 14 days in 100 nM folate (*n *=* *117); blue bars: BJ cells cultured for 14 days in 100 nM folate and supplemented with A, G, C, and T nucleosides for the last 48 h of the experiment (*n *=* *117). (E) Box plot summarizing the fork rate distribution (Kb/min) of three independent experiments. Control (*n *=* *352); 100 nM folate (*n *=* *364); 100 nM folate + AGCT (*n *=* *355). Main box represents the values from the lower to upper quartile (25^th^ to 75^th^ percentile). The middle line represents the median. (F) Fork distance (Kb) distribution. The color code is as in (D). Control (*n *=* *69); 100 nM folate (*n *=* *74); 100 nM folate + AGCT (*n *=* *72). (G) Box plot summarizing the fork distance distribution (Kb) of three independent experiments. Control (*n *=* *201); 100 nM folate (*n *=* *220); 100 nM folate + AGCT (*n *=* *228). Main box represents the values from the lower to upper quartile (25^th^ to 75^th^ percentile). The middle line represents the median. **P <* *0.001.

Next, we studied the effect of various folate concentrations on the DNA replication dynamic in cells grown for 14 and 21 days (Fig2B and C). On day 14, cells cultured at 100 nM, 20 nM, or in a folate-free medium exhibited a concentration-dependent decrease in the average fork rate and distance (Fig2B and C). Consistent with the above, the average fork rate and distance did not significantly differ between cells cultured with 500 nM folate and the control cells (Fig2B and C). The effect of folate deficiency on the average replication rate and fork distance significantly increased with time (Fig2B and C). Remarkably, cells grown in a medium with 500 nM folate, which did not affect cell proliferation (Fig2A), also showed a significant decrease in their average replication rate with time: After 14 days, the replication rate was 1.22 Kb/min (the same rate as in the control cells), whereas after 21 days the rate was significantly lower (Fig1B). The average fork distance in the 500 nM folate cultures decreased during this period of time from 127 to 97 Kb (Fig1C).

We further analyzed the effect of nucleoside supplementation on replication stress under mild folate deficiency. As can be seen in Fig2, BJ cells grown for 14 days in 100 nM folate showed a reduced replication rate (from 1.2 to 0.9 Kb/min) (*P* < 4.1 × 10^−10^) (Fig2B and D). Similar results were obtained in three independent experiments (Fig2E). In accordance with the reduced replication rate, the fork distance reduced from an average of 136 to 85 Kb (*P* < 2.3 × 10^−3^) (Fig2C and F). Similar results were obtained in three independent experiments (Fig2G). Supplementation of nucleosides for 48 h resulted in almost complete rescue of the average fork rate (*P* < 3.3 × 10^−9^) and distance (*P* < 0.005), (Fig2D–G). It is worth noting that the replication stress preceded the impaired proliferation, since cell growth for 14 days in 100 nM folate showed perturbed replication dynamics but no effect on cell proliferation. This indicates that the replication stress induced by folate deficiency was not secondary to decreased proliferation.

Altogether, our data show that the extent of replication stress is determined by folate deficiency in a concentration-dependent manner. Moreover, the effect of folate deficiency exacerbates with time, and even a mild chronic suboptimal folate level that does not hinder cell proliferation eventually results in stress on DNA replication.

### Enhanced replication stress and DNA damage in oncogene-expressing cells caused by folate deficiency

**Figure 3:**
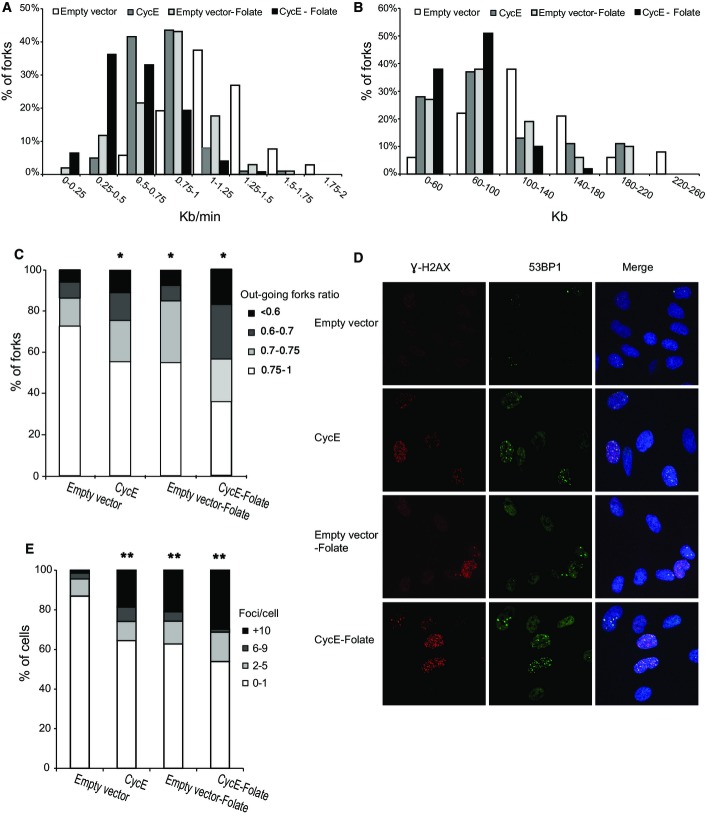
The effect of folate deficiency on replication dynamics and DSB formation in cyclin E-expressing cells Cyclin E-expressing BJ cells were grown for 7 days with and without folate.
Fork rate (Kb/min) distribution. White bars: BJ cells expressing an empty vector (*n* = 145); dark gray bars: BJ cells expressing the cyclin E oncogene (*n* = 147); light gray bars: BJ cells cultured for 7 days in a folate-free medium (*n* = 135); black bars: BJ cells expressing the cyclin E oncogene cultured for 7 days in a folate-free medium (*n* = 138).

Next, we studied whether the replication stress conferred by folate deficiency can enhance the replication stress induced by an oncogene. First, we expressed the oncogene cyclin E, which is frequently overexpressed in many types of human precancerous and cancerous lesions (Hwang & Clurman, 2005). Aberrant expression of cyclin E was shown to induce replication stress (Bester *et al*, 2011; Jones *et al*, 2013). Using retroviral infection, BJ cells were transfected with a cyclin E construct. Cyclin E expression was verified by Western blot analysis (Appendix Fig S3A). The experiments were performed in newly transformed cells, no later than 6 weeks following cyclin E infection. Cells were cultured for 7 days in a normal or folate-free medium. As can be seen in Fig3, folate deficiency significantly enhanced the replication stress conferred by cyclin E expression. Whereas cyclin E expression by itself decreased the average replication rate from 1.18 Kb/min in cells expressing an empty vector to 0.79 Kb/min (*P* < 2.4 × 10^−21^), folate deficiency further reduced the average replication rate to 0.59 Kb/min (*P* < 1 × 10^−13^) (Fig3A). The fraction of very slow replicating forks found in cyclin E-expressing cells was further increased when cells were cultured in a folate-free medium (Fig3A). Similarly, the average fork distance was further decreased when cyclin E-expressing cells were cultured in a folate-free medium, from 129 Kb in the control cells to 94 Kb in cyclin E-expressing cells (*P* < 8.4 × 10^−4^) and to 70 Kb in cyclin E-expressing cells grown in a folate-deficient medium (*P* < 1 × 10^−3^) (Fig3B). Similar results were obtained in three independent experiments (Appendix Fig S3B and C).

Fork distance distribution (Kb). The color code is as in (A). Empty vector (*n* = 78); CycE (*n* = 79); empty vector −folate (*n* = 71); CycE −folate (*n* = 80).

Percent of origins with the indicated progression ratio between sister forks. Empty vector (*n* = 158); CycE (*n* = 155); empty vector −folate (*n* = 160); CycE −folate (*n* = 154). *P <* *0.05.

Examples of nuclei with γH2AX and 53BP1 foci following cyclin E expression (CycE) (*n* = 65), empty vector (*n* = 65), folate-free medium for 7 days (empty vector −folate) (*n* = 67) or oncogene expression under folate-free conditions (CycE −folate) (*n* = 70). Red: γH2AX; green: 53BP1; blue: DAPI staining.

Percent of nuclei with the indicated number of γH2AX-53BP1 co-localized foci. **P <* *0.01. Data information: Bars represent average values.

Two replication forks that emerge from the same origin (sister forks) tend to exhibit the same replication rate (Anglana *et al*, 2003). However, under replication stress conditions, perturbed fork progression might lead to asymmetric progression of the sister forks (Di Micco *et al*, 2006). As previously suggested (Anglana *et al*, 2003), the progression of sister forks is considered symmetric when the ratio between them is > 0.75. Our analysis revealed a significant increase in the asymmetry between sister forks, from 23% in the control cells to 42% in cells grown under folate deficiency and 43% in cyclin E-expressing cells (Fig3C). Importantly, cyclin E-expressing cells grown under folate deficiency showed a further increase in the fraction of asymmetric forks to 67% (Fig3C). These results indicate that the replication perturbation induced by aberrant oncogene expression can be enhanced by an additional source of stress such as folate deficiency.

Next, we studied the effect of folate deficiency in cells expressing another oncogene, the human papilloma virus 16 (HPV16) E6/E7. In recent years, a correlation between folate deficiency and the development of HPV-induced cervical carcinoma has been reported (Rampersaud *et al*, 2002; García-Closas *et al*, 2005). We further investigated the effect of folate deficiency on replication dynamics in primary keratinocytes derived from adult skin biopsies expressing the HPV16 oncogenes E6/E7. This is a highly powerful model system for studying events in early stages of cervical cancer development, as primary keratinocytes are the natural host for HPV infection. All the experiments were performed in newly transformed cells 2–6 weeks following E6/E7 infection and before anaphase bridges and micronuclei were visible. Replication analysis was performed on E6/E7-expressing cells grown in a normal and a folate-free medium for 4 weeks. The average replication rate of the E6/E7-expressing keratinocytes in the normal medium was 0.79 Kb/min, whereas in the folate-free medium the average fork rate was significantly reduced to 0.58 Kb/min (*P* < 1.5 × 10^−5^) (Appendix Fig S4A), indicating that folate deficiency significantly enhances the effect of E6/E7 oncogenes on cellular DNA fork progression. We further studied the effect of folate deficiency on fork distance. We found that in E6/E7-expressing cells grown in a folate-free medium, the average fork distance was significantly shorter than in E6/E7-expressing cells grown in a normal medium (*P* < 5 × 10^−3^) (Appendix Fig S4B). Overall, our data show that the enhancement of oncogene-induced replication stress by folate deficiency is not oncogene or cell type specific.

We further studied the effect of folate deficiency on genome stability by analyzing the formation of DSBs (indicated by the γH2AX-53BP1 foci) in cyclin E-expressing cells grown for 7 days in a folate-free medium. Cyclin E-expressing cells cultured in the folate-free medium showed a significant increase in the number of γH2AX-53BP1 foci per nucleus compared to each treatment by itself (average of 7.8 and 4.6 foci/cell, respectively, Fig3D and E). In particular, the fraction of cells with a high level of γH2AX-53BP foci increased in cyclin E-expressing cells from 4% in the control cells to 25%. This fraction was further increased in cyclin E-expressing cells cultured in a folate-free medium, in which 32% of the nuclei showed a high level of γH2AX-53BP1 foci (Fig3D and E).

**Figure 4:**
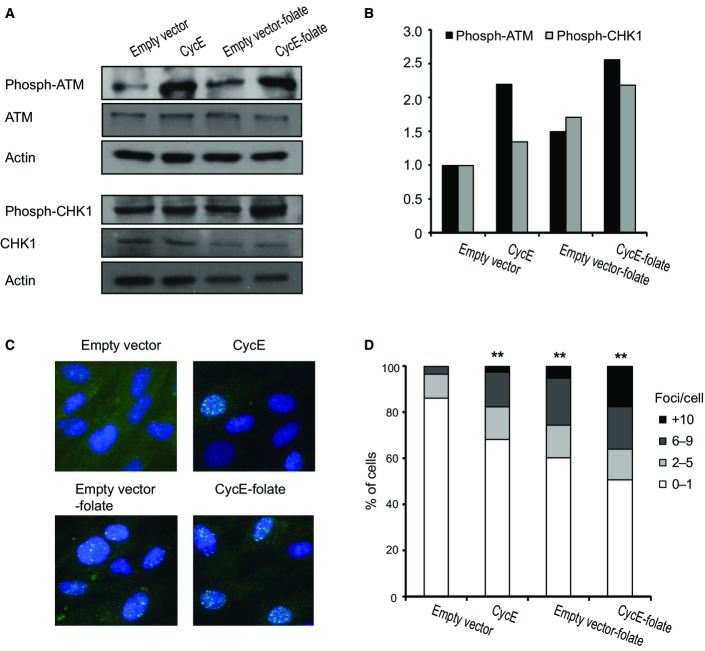
The effect of folate deficiency on DNA damage and signal transduction pathways Cyclin E-expressing BJ cells grown for 7 days with and without folate.
Immunoblotting with anti-phosphorylated ATM and anti-phosphorylated CHK1 antibodies. Anti-β-catenin and anti-actin antibodies were used as loading controls.

We further characterized the effect of folate deficiency on DNA damage signaling. For this, we studied the activation of the two main signal transduction pathways that inhibit cell-cycle progression following DNA damage, and the ATM and ATR pathways (Kastan & Bartek, 2004). The ATM protein is a member of the phosphatidylinositol 3-kinase family of proteins that respond to DNA damage by phosphorylating key substrates involved in DNA repair and/or cell-cycle control. The level of phosphorylated ATM was analyzed by Western blot analysis using an antibody against phosphorylated ATM (Fig4A and B). The results showed that cyclin E expression led to more than a twofold increase in the level of phosphorylated ATM. Folate deficiency by itself led to an increase of ∼1.5-fold in the level of phosphorylated ATM (Fig4A and B). Importantly, the combined effect resulted in more than a 2.5-fold increase in the level of phosphorylated ATM (Fig4A and B). Next, we studied the activation of the ATR pathway by analyzing the level of phosphorylated CHK1 which is increased under DNA damage, preferentially by ATR (Kastan & Bartek, 2004). As can be seen in Fig4A and B, both cyclin E expression and folate deficiency resulted in increased levels of phosphorylated CHK1. Importantly, in cyclin E-expressing cells grown under folate deficiency, the increase in the phosphorylated CHK1 level was higher than in each treatment by itself (Fig4A and B). Altogether, these results show that cyclin E expression and folate deficiency lead to the activation of both ATM and ATR signaling pathways, as found in other cellular stress responses (Kastan & Bartek, 2004). Importantly, the activation of both ATM and ATR signaling pathways was enhanced by the combination of oncogene expression and folate deficiency.

Protein level quantification.

Examples of nuclei with RAD51 foci following cyclin E expression (CycE) (*n* = 67), empty vector (*n* = 65), folate-free medium for 7 days (−folate) (*n* = 71) or oncogene expression under folate-free conditions (CycE −folate) (*n* = 75). Green: RAD51, blue: DAPI staining.

Percent of nuclei with the indicated number of RAD51 foci (*n* = 66). **P <* *0.01.

Next, we studied RAD51 foci formation in response to folate deficiency. *RAD51* plays a critical role in homologous recombination and therefore in DSB repair (Petermann *et al*, 2010). Furthermore, *RAD51* was recently shown to be essential for replication fork reversal and restart upon different types of replication stress conditions (Zellweger *et al*, 2015). As can be seen in Fig4C and D, cyclin E-expressing cells cultured in a folate-free medium showed a significant increase in the number of RAD51 foci per nucleus compared to the number in each treatment by itself. The fraction of cells with RAD51 foci increased in cyclin E-expressing cells from 15% in the control cells to 35% (Fig4C and D). This fraction was further increased in cyclin E-expressing cells cultured in a folate-free medium, in which almost half of the nuclei showed RAD51 foci (Fig4C and D). Altogether, these results indicate that the extent of oncogene-induced replication stress can be enhanced by an additional source of stress, resulting in enhanced DNA damage.

### Enhanced tumorigenicity in oncogene-expressing cells caused by folate deficiency both *in vitro* and *in vivo*

**Figure 5:**
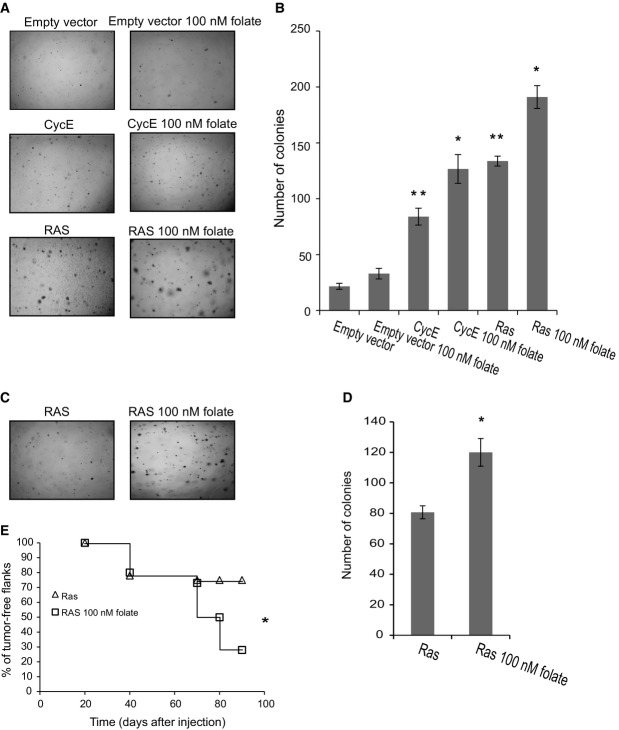
The effect of folate deficiency on the tumorigenicity of oncogene-expressing cells *in vitro* and *in vivo* A, B Cyclin E- and *Ras* (*H-RasV12*)-expressing 3T3 cells grown in 100 nM folate for 4 weeks and then two additional weeks in a normal medium. Control cells were grown in a normal medium for the whole period. (A) Examples of anchorage-independent growth in soft agar of 3T3 cells. (B) Average number of colonies per soft agar plate of 3T3 cells. The number of colonies per plate is expressed as the average ± SEM from three independent experiments.

We next investigated whether the enhanced genomic instability caused by folate deficiency enhances cancer development. For this purpose, we performed a standard *in vitro* transformation assay that measures anchorage-independent growth in soft agar in both mouse and human cells. We analyzed the colony-forming capacity of mouse 3T3 cells expressing either the human cyclin E or the oncogenic *Ras* (*H-RasV12*). Cells were grown for 4 weeks in a normal medium or in a mild folate-deficient medium (100 nM) and then for 2 more weeks in a normal medium, to allow recovery of the cells from proliferation arrest due to the prolonged growth in folate-deficient conditions. This enabled evaluation of the tumorigenicity potential of the cells due to the folate deficiency-induced DNA damage. Mild folate deficiency by itself did not affect the colony-forming capacity of the cells (Fig5A and B). However, mild folate deficiency significantly increased colony formation caused by oncogene expression from an average of 84 colonies per plate in the 3T3 cyclin E-expressing cells grown in a normal medium to 127 per plate in the 3T3 cyclin E-expressing cells grown under mild folate deficiency conditions (*P* < 0.05) (Fig5A and B). Similar results were found following the expression of the oncogene *Ras*. Activating mutations in *Ras* such as G12V are found in many human cancers (Karnoub & Weinberg, 2008), and lead to DSBs that result in structural as well as numerical instability (Denko *et al*, 1994; Spruck *et al*, 1999; Abulaiti *et al*, 2006). Our analysis showed that *Ras* expression by itself significantly induced colony formation from 22 colonies per plate in the control cells to 134 in the *Ras*-expressing cells (*P* < 0.01) (Fig5A and B). Similar to the effect of folate deficiency on cyclin E-expressing cells, mild folate deficiency significantly increased colony formation in the *Ras*-expressing cells from 134 to 191 per plate in cells grown in the mild folate-deficient medium (*P* < 0.05) (Fig5A and B). It is important to note that 3T3 cells grown in a medium with a severe folate deficiency (20 nM folate) or in a folate-free medium stopped growing within 2 weeks, with or without the expression of cyclin E or *Ras*.

C–E *Ras* (*H-RasV12*)-expressing MCF10A cells grown in 100 nM folate for 4 weeks and then two additional weeks in a normal medium. Control cells were grown in a normal medium for the whole period. (C) Examples of anchorage-independent growth in soft agar of MCF10A cells. (D) Average number of colonies per soft agar plate of MCF10A cells. The number of colonies per plate is expressed as the average ± SEM from three independent experiments. (E) Percentage of tumor-free flanks at the indicated time points after cell injection. Ten mice were injected in both sides in each group. Data information: Cells expressing pBABE (Empty vector); cells expressing an empty vector and grown in 100 nM folate (pBABE 100 nM Folate); cells expressing the cyclin E oncogene (CycE); cells expressing the cyclin E oncogene and grown in 100 nM folate deficiency (CycE 100 nM folate); cells expressing the *Ras* oncogene (Ras); cells expressing the *Ras* oncogene and grown in a 100 nM folate-deficient medium (Ras 100 nM Folate). *P <* *0.05, **P <* *0.01.

Next, we studied the effect of folate deficiency on colony formation in immortal human cells aberrantly expressing the oncogenic *Ras*. We used immortal human breast MCF10A cells transformed by oncogenic *Ras,* grown for 4 weeks in a normal medium or in a mild folate-deficient medium (100 nM folate) and for an additional 2 weeks in a normal medium. The results showed that folate deficiency significantly increased colony formation caused by *Ras* expression from 81 colonies per well in MCF10A-*Ras*-expressing cells grown in a normal medium to 120 in MCF10A-*Ras*-expressing cells grown in mild folate deficiency conditions (Fig5C and D). These results indicate that the *in vitro* tumorigenic potential of cells aberrantly expressing an oncogene is significantly enhanced by mild folate deficiency.

We further investigated the effect of folate deficiency in oncogene-expressing cells on tumor development *in vivo*. For this purpose, we injected *Ras*-transformed MCF10A cells, grown 4 weeks in a normal medium or in a mild folate-deficient medium (100 nM folate) and for an additional 2 weeks in a normal medium into (Atimic-Nu/Nu) nude mice. The results showed that in mice injected with MCF10A-*Ras* cells grown in a folate-deficient medium, the percentage of developed tumors was significantly higher than in those mice injected with cells grown in a normal medium (72 and 28%, respectively) (Fig5E). These results clearly demonstrate that folate deficiency significantly enhances tumor development caused by oncogene expression *in vivo*.

## Discussion

Here, we show that the extent of replication stress plays an important role in prompting genomic instability and tumor development *in vivo* (Figs5). Our results indicate that replication-induced genome instability and tumorigenicity can be induced by both genetic and non-genetic (e.g., dietary) factors. We found that micronutrients such as folate can significantly enhance the replication stress caused by oncogene expression and therefore reinforce cancerous processes (Figs5). Strikingly, the percentage and not the size of the developing tumors was significantly higher when oncogene-expressing cells were grown under folate-deficient conditions. This suggests that the effect of folate deficiency on tumorigenicity cannot be merely explained by its effect on cell proliferation but rather by acting as an additional driving force enhancing the oncogene-induced transformation.

Notably, enhanced tumorigenicity both *in vitro* and *in vivo* was found after injection of cells that were allowed to recover for several passages after the folate deficiency regime. This implies that even a transient folate deficiency is sufficient to disrupt genome integrity and enhance tumorigenicity, as DNA damage that was generated under conditions of folate deficiency is irreversible and thus cannot be recovered subsequent to later folate supplementation. Altogether, our results show that *in vivo* development of cancer is mediated by a combination of genetic and non-genetic factors that affects the extent of replication-induced genomic instability.

Diet is estimated to contribute to about one-third of preventable cancers (reviewed in (Ames & Wakimoto, 2002), but the mechanisms by which dietary micronutrients promote DNA damage and carcinogenesis are not fully understood. The principal mechanism linking folate deficiency to DNA damage is thought to be the incorporation of dUMP into the DNA (Blount *et al*, 1997). Here, we showed that folate deficiency affects genome stability even earlier, as it perturbs the replication dynamics that lead to replication stress-induced genome instability.

Recently, growth under folate-free conditions was shown to increase the frequency of HPV16 infections and the transformation of HPV16-infected tissues (Xiao *et al*, 2012). The proposed mechanism in that study was alteration in cellular–viral protein interactions, due to activation of a nutrition-sensitive posttranscriptional RNA operon. Our work, however, suggests a general mechanism for the effect of folate in oncogene-expressing cells, by showing that folate deficiency in both cellular and viral oncogene-expressing cells (BJ cells expressing cyclin E and keratinocytes expressing HPV16 E6/E7 oncogenes, respectively) enhances DNA replication stress, resulting in increased genomic instability and tumorigenicity (Figs5; Appendix Figs S3 and S4).

Acute deficiencies of vitamins and minerals are rare in developed countries; however, suboptimal intake is a widespread problem that can lead to considerable cellular damage (Ames & Wakimoto, 2002). Our data show a concentration-dependent effect of folate deficiency on replication dynamics. Interestingly, even a very mild deficiency reduced the replication rate and fork distance over time (Fig2), demonstrating that a mild (suboptimal) but chronic folate deficiency might be extremely significant in association with genetic changes in cancer genes.

It would be valuable to relate the *in vitro* values to physiological values. This is extremely challenging, primarily because folate is supplemented in tissue culture media as folic acid while *in vivo* it is provided through nutrition in the form of various folate derivatives, whose cellular uptake is much more efficient than the uptake efficiency of folic acid. Moreover, differences among individuals in the efficiency to absorb and metabolize this vitamin (reviewed in Fenech, 2012) also affect the actual folate level *in vivo*. Further epidemiological, clinical, and interventional studies are required to determine the physiological levels of folate deficiency and the deficiency duration that affect replication dynamics.

The proliferation of normal primary cells was arrested under prolonged mild or severe folate deficiency (0–100 nM) (Figs1 and 2). During this period, the cells accumulated replication stress leading to genome instability. In the same cells expressing an oncogene, the effect of folate deficiency significantly enhanced the replication stress and genome instability induced by the oncogene (Figs3 and 4). When folate levels returned to normal, the oncogene-expressing cells showed a significantly higher tumorigenic potential compared to the potential of their counterparts grown under normal conditions (Fig5). These results show that cells expressing an oncogene for a short time have increased sensitivity to folate deficiency than both normal cells and oncogene-expressing cells grown under folate deficiency.

Furthermore, these results may explain the development of secondary malignancies following antifolate drug treatment, as the drug may promote their transformation. A better understanding of the effects of antifolate drugs, on the mechanisms that initiate, direct, and enable chromosomal instability is of major clinical importance and might lead to the development of better therapeutic approaches. An additional well-established phenomenon hindering the therapeutic potential of antifolate drugs is antifolate resistance that is frequently developed by several molecular mechanisms such as qualitative and/or quantitative alterations in influx and/or efflux transporters of antifolates and in folate-dependent enzymes (Assaraf, 2007; Gonen & Assaraf, 2012). Indeed, this has been our rationale to establish a modal system that mimics folate deficiency based on folate-deficient medium rather than antifolate drugs, and mimics more accurately the gene–nutrition interactions early in cancer development.

Replication stress is considered to be a complex phenomenon that has severe implications for genome stability, cell survival, and human disease. We used folate deficiency as a model to demonstrate the co-carcinogenic interaction between dietary and genetic factors that is mediated by their effect on the DNA replication machinery. It is widely accepted that the initiation of cancer is a result of a combination of multiple genetic alterations, referred to as hits. Our results suggest that folate deficiency functions as a non-genetic hit which in conjunction with oncogene expression can enforce the cancerous process. Hence, replication stress is a quantitative trait that serves as a molecular mechanism linking oncogene expression, folate deficiency and cancer development.

## Materials and Methods

### Cell cultures

Primary human diploid foreskin fibroblasts (BJ cells) expressing a transfected hTERT (Bodnar *et al*, 1998) were grown in folate-free DMEM (custom-made, Biological Industries, Beit Haemek, Israel) or normal DMEM (Biological Industries, Beit Haemek, Israel). These concentrations were estimated by the manufacturer. The medium was supplemented with 5% FBS, 100,000 U/l penicillin, and 100 μg/l streptomycin. For different folate concentrations, folate-free DMEM and normal DMEM (containing 9,040 nM folate) were mixed in the desired ratios. Primary keratenocytes expressing transfected E6/E7 were grown in folate-free RPMI (custom-made, Biological Industries, Beit Haemek, Israel) or normal RPMI (Biological Industries, Beit Haemek, Israel) supplemented with 5% FBS, 100,000 U/l penicillin, and 100 μg/l streptomycin. Mouse immortalized fibroblasts 3T3 cells transfected with cyclin E or *Ras* (*H-RasV12*) and mammary epithelial cells MCF10A transfected with *Ras* (*H-RasV12*) were grown for 4 weeks in a DMEM that contained 100 nM folate (1:90.4 ratio between normal DMEM and folate-free DMEM, respectively) (Biological Industries, Beit Haemek, Israel) supplemented with 5% FBS, 100,000 U/l penicillin, and 100 μg/l streptomycin. Then, they were maintained two additional weeks in a normal DMEM (Biological Industries, Beit Haemek, Israel) supplemented with 5% FBS, 100,000 U/l penicillin, and 100 μg/l streptomycin. Control cells were grown in a normal medium for the whole period.

### Retroviral infection

Amphotropic retroviruses expressing cyclin E or *Ras* (*H-RasV12*) were generated by Phoenix retroviral packaging cells according to established procedures. The cyclin E pBABE-puro-based vector was kindly provided by Professor J. Bartek. The phoenix cells were grown in DMEM supplemented with 10% fetal bovine serum (FBS), and the supernatant was collected. hTERT-expressing BJ cells 3T3 and MCF10A cells were infected according to established procedures. Primary keratenocytes were infected as was described previously (Bester *et al*, 2011).

### Molecular combing

Unsynchronized cells were pulse-labeled for 30 min by a medium containing 100 μM of the thymidine analog iododeoxyuridine (IdU). At the end of the first labeling period, the cells were washed twice with a warm medium and pulse-labeled once more for 30 min with a medium containing 100 μM of another thymidine analog chlorodeoxyuridine (CldU). Cells were then harvested, and genomic DNA was extracted, combed, and analyzed as previously described (Lebofsky *et al*, 2006; Herrick & Bensimon, 2009). The primary antibody for fluorescence detection of IdU was mouse anti-BrdU (Becton Dickinson), and the secondary antibody was goat anti-mouse Alexa Fluor 488 (Invitrogen). The primary antibody for fluorescence detection of CldU was rat anti-CldU (Novus Biologicals). The secondary antibody was goat anti-rat Alexa Fluor 594 (Invitrogen). The length of the replication signals and the distances between origins were measured in micrometers and converted to kilo bases according to a constant and sequence-independent stretching factor (1 μm = 2 Kb), as previously reported (Herrick & Bensimon, 2009).

### Nucleoside supplementation

The ribonucleosides adenosine, cytidine, guanosine (Sigma), and deoxyribonucleoside thymidine (Sigma) were freshly prepared for each experiment, filter-sterilized, and used at 50 μM each in the last 48 h of the experiment.

### Soft agar assay

Following cyclin E and *Ras* (*H-RasV12*) expression in 3T3 and *Ras* (*H-RasV12*) expression in MCF10A, cells were grown in a DMEM that contained 100 nM folate for 4 weeks and then two additional weeks in a normal medium. Control cells were grown in a normal medium for the whole period. 4 × 10^3^ cells in a growth medium containing 0.3% agar (50 *μ*l final volume) were plated on the top of a solid growth medium containing 1% agar (100 μl final volume). After the medium solidified, an additional 50 μl of growth medium was added (Agarose, Type VII, Sigma). Colonies were counted after 4–6 weeks of growth.

### Animal care

All animal experiments were performed in accordance with the guidelines of the Hebrew University Committee for the use of animals for research. Veterinary care was provided to all animals by the Hebrew University animal care facility staff in accordance with AAALAC standard procedures and as approved by the Hebrew University Ethics Committee.

### Tumorigenesis assays in nude mice

MCF10A cells expressing *H-RasV12* were grown in a DMEM that contained 100 nM folate for 4 weeks and then two additional weeks in a normal medium. Control cells were grown in a normal medium for the whole period. Cells were injected (3 × 10^6^ cells per site in 200 μl of PBS) subcutaneously into each rear flank of 8-week-old female (Atimic-Nu/Nu) nude mice by using a 26-gauge needle. Tumor growth was monitored every 10 days. Blinding and randomization have not been used.

### Immunofluorescence for detection of γH2AX, 53BP1, and RAD51 foci

BJ cells were fixed in 3.7% formaldehyde/PBS for 10 min, permeabilized with 0.5% Triton/PBS, and blocked with 5% BSA/PBS. The primary antibodies used were mouse anti-γH2AX 1:200 (Upstate Biotechnology), rabbit polyclonal anti-53BP1 1:200 (Bethyl Laboratories), and rabbit polyclonal anti-RAD51 1:200 (EMD MILLIPORE). Appropriate secondary antibodies were added (Jackson ImmunoResearch Laboratories). Images were taken with a Bio-Rad confocal microscope. For focus information analysis, at least 65 nuclei for each condition were analyzed.

### Western blotting

8–10% polyacrylamide gels were used for protein separation and detection. The gels were transferred to a nitrocellulose membrane, and antibody hybridization and electrochemiluminescence (ECL) were performed according to standard procedures. The primary antibodies used in this analysis were rabbit monoclonal anti-phosphorylated ATM 1:1,000 (Abcam), mouse monoclonal anti-ATM 1:10,000 (sigma), rabbit monoclonal anti-phosphorylated CHK1 1:200 (Cell Signaling), mouse monoclonal anti-CHK1 1:500 (Abcam), mouse monoclonal anti-cyclin E 1:200 (Santa Cruz Biotechnology), and rabbit polyclonal anti-beta actin 1:5,000 (Abcam). HRP-conjugated anti-rabbit and anti-mouse secondary antibodies were obtained from Jackson ImmunoResearch laboratories (West Grove, PA).

### Nucleotide pool analysis

Cells were harvested, and cellular nucleotides were extracted with 0.4 N perchloric acid and neutralized with potassium chloride. Deoxynucleotides were separated from ribonucleotides using a boronate affinity column. Deoxynucleotides were analyzed by HPLC using UV absorbance at 254 and 281 nm for identification and quantitation as previously described (Flanagan *et al*, 2007).

### Statistics

A summary of the number of repeats for each experiment and the exact *P*-value and the statistical tests that have been employed can be found in Appendix Table S1. In summary, replication dynamics experiments, immunofluorescence experiments, and soft agar experiments were performed at least three independent times. Two-tailed Student’s *t*-tests were performed to determine significant differences between treatment groups. Fisher’s exact test was performed to determine significant differences in the symmetry of fork progression. To determine overall tumor development in mice, we used a log-rank (Mantel–Cox) test. Quantitative analyses were conducted blindly.

## Supporting Information

**Appendix**

**Review Process File**
