# Supplementary material for: Folate levels modulate oncogene-induced replication stress and tumorigenicity
Source: EMBO Mol Med. 2015 Jul 21;7(9):1138–52. doi: 10.15252/emmm.201404824 (PMC4568948; doi:10.15252/emmm.201404824)
Supplement: Supplementary file 1 [file emmm0007-1138-sd1.pdf]

Table S1: A summary of experimental design

Method	Description	# of repeats	Repeats presented	Figure no.	Sample size	Statistic tests and p-values
Population doublings (PDs)	BJ cells were cultured for 48 days under different folate concentration	3	1	1A, 2A	NA	NA
DNA combing 1	BJ cells were cultured for 7 days with and without folate and nucleosides. Replication rate was measured	3	2 and a summary of the three repeats	Repeat 1- 1D	n=number of fibers<br>1. Control (n=126)<br>2. -Folate (n=131)<br>3. -Folate + AGCT (n=138)	Two tailed t-test<br>1-2 p= 1.6×10^{-32}<br>2-3 p= 3×10^{-31}
				Repeat 2- S1A	n=number of fibers<br>1. Control (n=151)<br>2. -Folate (n=149)<br>3. -Folate + AGCT (n=145)	Two tailed t-test<br>1-2 p= 4.2×10^{-30}<br>2-3 p= 2.8×10^{-28}
				Summary- 1E	n=number of fibers<br>1. Control (n=360)<br>2. -Folate (n=372)<br>3. -Folate + AGCT (n=361)	Two tailed t-test<br>1-2 p= 5.5×10^{-42}<br>2-3 p= 6.8×10^{-39}
	BJ cells were cultured for 7 days with and without folate and nucleosides. Fork distance was measured	3	2 and a summary of the three repeats	Repeat 1- 1F	n=number of forks<br>1. Control (n=72)<br>2. -Folate (n=71)<br>3. -Folate + AGCT (n=75)	Two tailed t-test<br>1-2 p= 4×10^{-11}<br>2-3 p= 2.5×10^{-7}
				Repeat 2- S1B	n=number of forks<br>1. Control (n=78)<br>2. -Folate (n=78)<br>3. -Folate + AGCT (n=80)	Two tailed t-test<br>1-2 p= 1.1×10^{-7}<br>2-3 p= 3.2×10^{-5}
				Summary- 1G	n=number of forks<br>1. Control (n=78)<br>2. -Folate (n=78)<br>3. -Folate + AGCT (n=80)	Two tailed t-test<br>1-2 p= 2.6×10^{-14}<br>2-3 p= 3.7×10^{-9}
DNA combing 2	BJ cells were cultured for 14 days under different folate concentrations. Replication rate was measured.	2	1	2B	n=number of fibers<br>1. 0 nM (n=115)<br>2. 20 nM (n=132)<br>3. 100 nM (n=125)<br>4. 500 nM (n=128)<br>5. Normal DMEM (n=129)	Two tailed t-test<br>1-5 p= 7.3×10^{-22}<br>2-5 p= 5.7×10^{-19}<br>3-5 p= 1.8×10^{-10}<br>4-5 p= non significant
	BJ cells were cultured for 14 days under different folate concentrations. Fork distance was measured	2	1	2C	n=number of forks<br>1. 0 nM (n=71)<br>2. 20 nM (n=81)<br>3. 100 nM (n=74)<br>4. 500 nM (n=80)<br>5. Normal DMEM (n=83)	Two tailed t-test<br>1-5 p= 9.3×10^{-9}<br>2-5 p= 8.3×10^{-7}<br>3-5 p= 4.5×10^{-4}<br>4-5 p= non significant
DNA combing 3	BJ cells were cultured for 21 days under different folate concentrations. Replication rate was measured.	2	1	2B	n=number of fibers<br>1. 0 nM (n=117)<br>2. 20 nM (n=117)<br>3. 100 nM (n=115)<br>4. 500 nM (n=118)<br>5. Normal DMEM (n=131)	Two tailed t-test<br>1-5 p= 6.5×10^{-24}<br>2-5 p= 7.3×10^{-22}<br>3-5 p= 3.8×10^{-11}<br>4-5 p= 4.7×10^{-7}
	BJ cells were cultured for 21 days under different folate concentrations. Fork distance was measured	2	1	2C	n=number of forks<br>1. 0 nM (n=72)<br>2. 20 nM (n=74)<br>3. 100 nM (n=71)<br>4. 500 nM (n=75)<br>5. Normal DMEM (n=82)	Two tailed t-test<br>1-5 p= 1.2×10^{-9}<br>2-5 p= 5.4×10^{-8}<br>3-5 p= 1.7×10^{-5}<br>4-5 p= 1.3×10^{-4}

DNA combing 4	BJ cells were cultured for 14 days in 100nM folate with and without nucleosides. Replication rate was measured	3	1 and a summary of the three repeats	Repeat 1- 2D	n=number of fibers<br>1. Control (n=115)<br>2. 100 nM Folate (n=117)<br>3. 100 nM Folate + AGCT (n=117)	Two tailed t-test<br>1-2 p= 4.1×10^{-10}<br>2-3 p= 3.3×10^{-9}
				Summary- 2E	n=number of fibers<br>1. Control (n=352)<br>2. 100 nM Folate (n=364)<br>3. 100 nM Folate + AGCT (n=355)	Two tailed t-test<br>1-2 p= 3.7×10^{-11}<br>2-3 p= 5×10^{-8}
	BJ cells were cultured for 14 days in 100nM folate with and without nucleosides. Fork distance was measured	3	1 and a summary of the three repeats	Repeat 1- 2F	n=number of forks<br>1. Control (n=69)<br>2. 100 nM Folate (n=74)<br>3. 100 nM Folate + AGCT (n=72)	Two tailed t-test<br>1-2 p=2.3×10^{-3}<br>2-3 p= 5×10^{-3}
				Summary- 2G	n=number of forks<br>1. Control (n=201)<br>2. 100 nM Folate (n=220)<br>3. 100 nM Folate + AGCT (n=228)	Two tailed t-test<br>1-2 p= 7.8×10^{-4}<br>2-3 p= 9.1×10^{-3}
DNA combing 5	Cyclin E-expressing BJ cells were cultured for 7 days with and without folate. Replication rate was measured	3	2	Repeat 1- 3A	n=number of fibers<br>1. Empty vector (n=145)<br>2. CycE (n=147)<br>3. Empty vector – Folate (n=135)<br>4. CycE – Folate (n=138)	Two tailed t-test<br>1-2 p= 2.4×10^{-21}<br>1-3 p= 3.1×10^{-22}<br>2-4 p= 1×10^{-13}
				Repeat 2- S3B	n=number of fibers<br>1. Empty vector (n=136)<br>2. CycE (n=100)<br>3. Empty vector – Folate (n=115)<br>4. CycE – Folate (n=134)	Two tailed t-test<br>1-2 p= 3.6×10^{-20}<br>1-3 p= 2.8×10^{-18}<br>2-4 p= 5.1×10^{-10}
	Cyclin E-expressing BJ cells were cultured for 7 days with and without folate. Fork distance was measured	3	2	Repeat 1- 3B	n=number of forks<br>1. Empty vector (n=78)<br>2. CycE (n=79)<br>3. Empty vector – Folate (n=71)<br>4. CycE – Folate (n=80)	Two tailed t-test<br>1-2 p= 8.4×10^{-4}<br>1-3 p= 7.5×10^{-5}<br>2-4 p= 1×10^{-3}
				Repeat 2- S3C	n=number of forks<br>1. Empty vector (n=75)<br>2. CycE (n=55)<br>3. Empty vector – Folate (n=66)<br>4. CycE – Folate (n=72)	Two tailed t-test<br>1-2 p= 4×10^{-6}<br>1-3 p= 3.1×10^{-5}<br>2-4 p= 2.8×10^{-3}
	Cyclin E-expressing BJ cells were cultured for 7 days with and without folate. Fork symmetry was measured	3	Summary of three	Summary- 3C	n=number of forks<br>1. Empty vector (n=158)<br>2. CycE (n=155)<br>3. Empty vector – Folate (n=160)<br>4. CycE – Folate (n=154)	Two tailed t-test<br>1-2 p= 3.6×10^{-4}<br>1-3 p= 2.7×10^{-3}<br>2-4 p= 3.7×10^{-2}
DNA combing 6	keratinocytes expressing E6/E7cultured for 4 weeks with and without folate. Replication rate was measured	3	1	S4A	n=number of fibers<br>1. E6/E7 (n=151)<br>2. E6/E7 - Folate (n=167)	Two tailed t-test<br>1-2 p= 1.5×10^{-5}
	keratinocytes expressing E6/E7cultured for 4 weeks with and without folate. Fork distance was measured	3	1	S4B	n=number of forks<br>1. E6/E7 (n=85)<br>2. E6/E7 - Folate (n=93)	Two tailed t-test<br>1-2 p= 5×10^{-3}
IF 1	Cyclin E-expressing BJ cells were cultured for 7 days with and without folate. γH2AX-53BP1 co-localized foci were counted	3	1	3D and E	n=number of nuclei<br>1. Empty vector (n=65)<br>2. CycE (n=65)<br>3. Empty vector – Folate (n=67)<br>4. CycE – Folate (n=70)	Fisher's exact test<br>1-2 p= 4.2×10^{-3}<br>1-3 p= 8.6×10^{-3}<br>2-4 p= 9.5×10^{-3}

IF 2	Cyclin E-expressing BJ cells were cultured for 7 days with and without folate. RAD51 foci were counted	2	1	4C and D	n=number of nuclei<br>1. Empty vector (n=67)<br>2. CycE (n=65)<br>3. Empty vector – Folate (n=71)<br>4. CycE – Folate (n=75)	Fisher's exact test<br>1-2 p= 3.8×10^{-4}<br>1-3 p= 7.5×10^{-4}<br>2-4 p= 9.2×10^{-3}
Western Blot	Cyclin E-expressing BJ cells were cultured for 7 days with and without folate. Phosphorylated ATM and a CHK1 levels were measured	3	1	4A and B	NA	NA
Colony forming assay 1	*Cyclin E*-expressing 3T3 cells grown in 100nM folate for 4 weeks and then two additional weeks in a normal medium. The number of colonies per soft agar plate were counted	3	Summary of three	5A and B	n=number of plates<br><br>n= 3 plates for each condition	Two tailed t-test<br>Empty vector – CycE<br>p= 4.1×10^{-3}<br>CycE 100 nM folate–CycE<br>p= 3.6×10^{-2}
Colony forming assay 2	*Ras (H-RasV12)*- expressing 3T3 cells grown in 100nM folate for 4 weeks and then two additional weeks in a normal medium. The number of colonies per soft agar plate were counted	3	Summary of three	5A and B	n=number of plates<br><br>n= 3 plates for each condition	Two tailed t-test<br>Empty vector – Ras<br>p= 2.8×10^{-3}<br>Ras 100 nM folate–Ras<br>p= 1.2×10^{-2}
Colony forming assay 3	*Ras* (*H-RasV12*)-expressing MCF10A cells, grown in 100nM folate for 4 weeks and then two additional weeks in a normal medium. The number of colonies per soft agar plate were counted	3	Summary of three	5C and D	n=number of plates<br><br>n= 3 plates for each condition	Two tailed t-test<br>p= 1.1×10^{-2}
Tumorigenesis assay in nude mice	*Ras* (*H-RasV12*)-expressing MCF10A cells grown in 100nM folate for 4 weeks and then two additional weeks in a normal medium. Cells were injected subcutaneously into each rear flank of (Atimic-Nu/Nu) nude mice. The number and size of tumors were measured	1	1	5F	n=number of flanks<br><br>n=20 flanks(10 mice)	log-rank (Mantel-Cox) test<br>p= 3.1×10^{-2}
HPLC nucleotide pool analysis	Primary keratinocytes grown 15-30 days in folate-free medium. Nucleotide levels were measured	3	Summary of three	S2	NA	Two tailed t-test<br>p= 2.6×10^{-2}

Figure S1

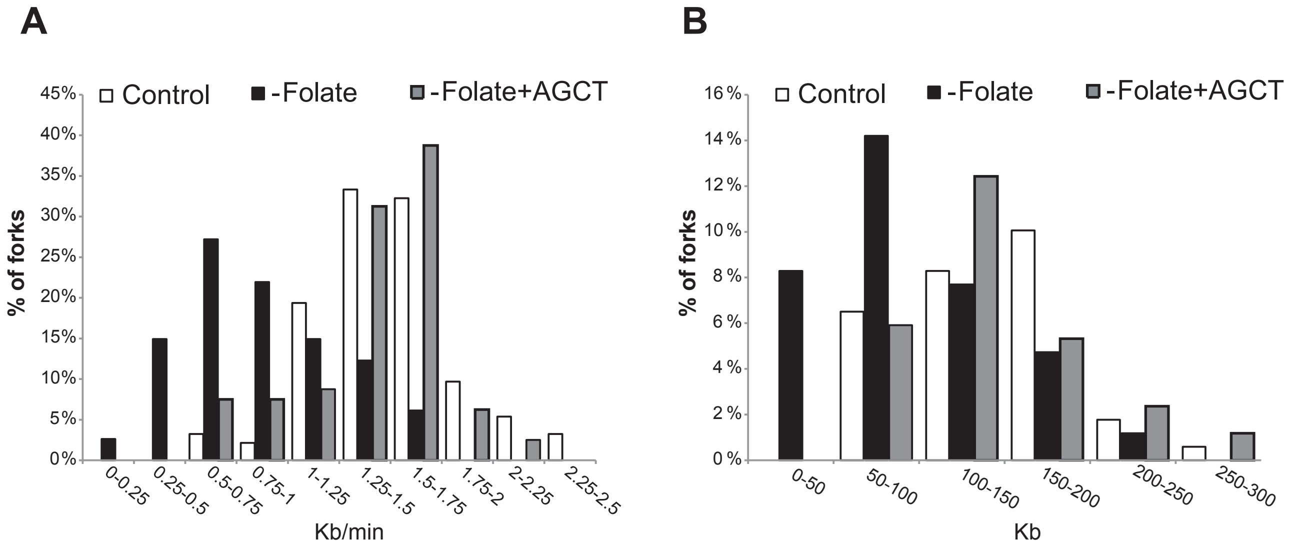

Figure S1. Growth rate and replication dynamics in BJ cells grown in a folate free-medium with and without nucleoside supplementation.

(A) Fork rate (Kb/min) distribution. (B) Fork distance (Kb) distribution. White bars - BJ cells; black bars - BJ cells that were cultured for 7 days in a folate- free medium; grey bars - BJ cells cultured for 7 days in folate-free medium and supplemented with A, G, C and T nucleosides for the last 48 hrs. of the experiment.

Figure S2

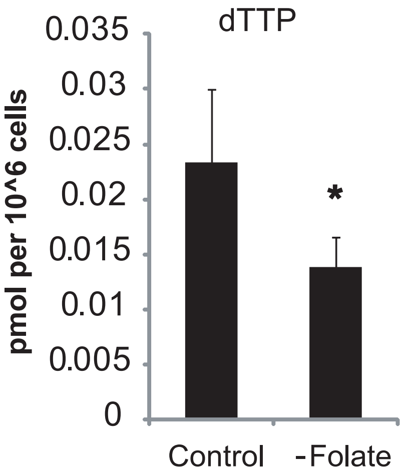

Figure S2. The effect of folate deficiency on the nucleotide pool. dTTP level in primary keratinocytes grown 15-30 days in folate-free medium. The levels are expressed as mean fold change ± SEM from three independent experiments.
*$p < 0.05$.

Figure S3

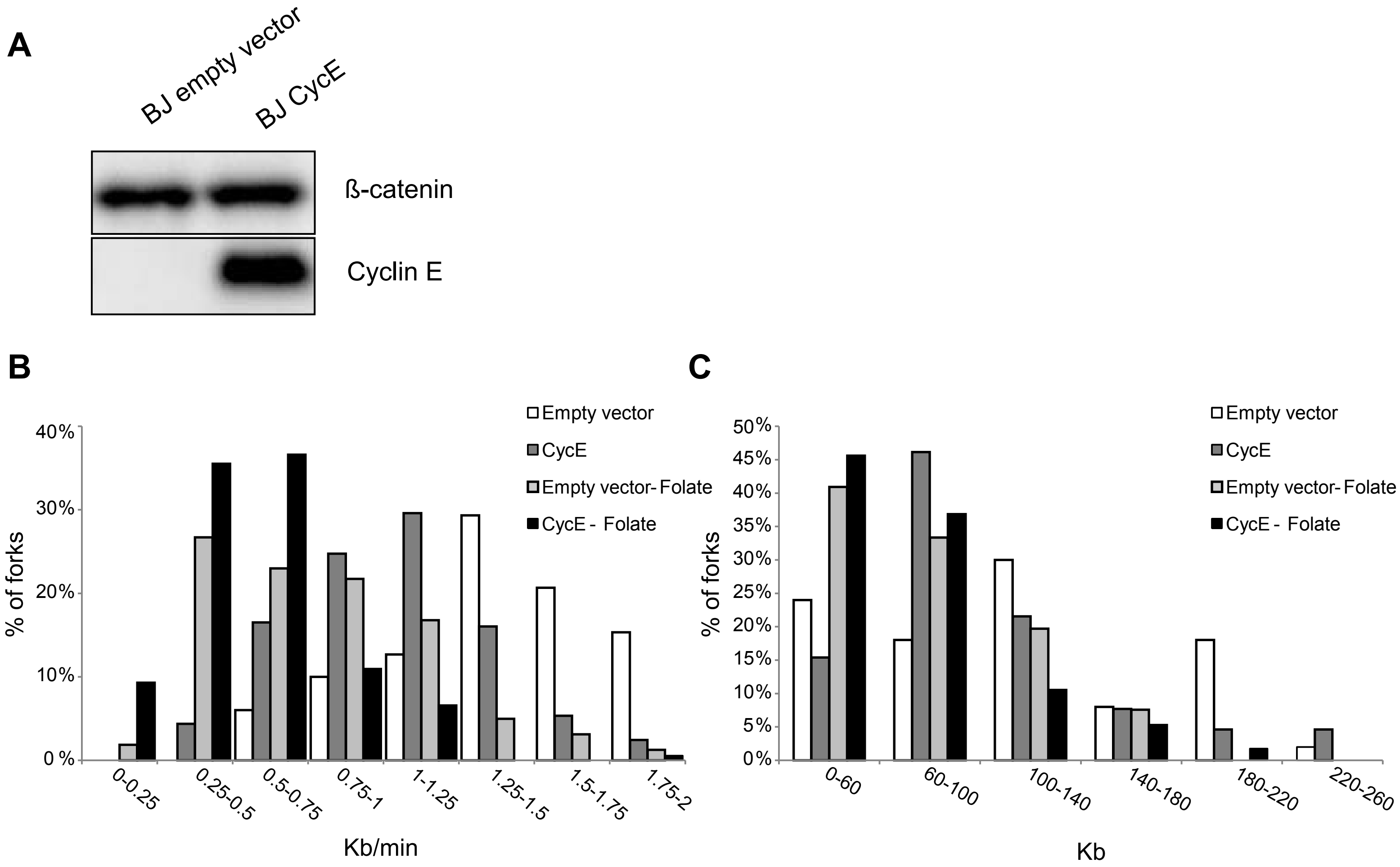

Figure S3. The effect of folate deficiency on replication dynamics in cyclin E- expressing cells. (A) Western blot analysis of Cyclin E protein levels in BJ cells expressing cyclin E or an empty vector. (B) Fork rate (Kb/min) distribution. (C) Fork distance (Kb) distribution. White bars - BJ cells expressing an empty vector; dark grey bars - BJ cells expressing the cyclin E oncogene; light grey bars - BJ cells cultured for 7 days in a folate-free medium; black bars - BJ cells expressing the cyclin E oncogene cultured for 7 days in a folate-free medium.

Figure S4

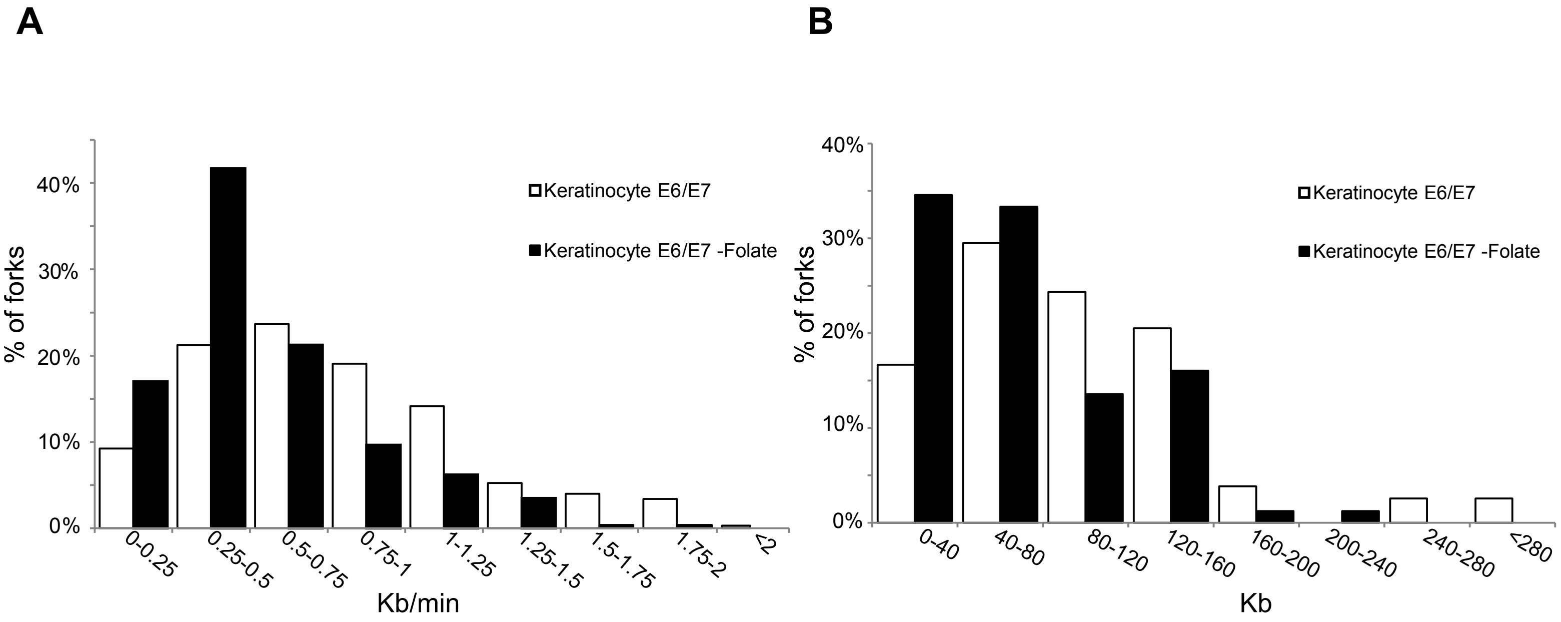

Figure S4. The effect of folate deficiency on replication dynamics in keratinocytes expressing the HPV- E6/E7 proteins. (A) Fork rate (Kb/min) distribution. (B) Fork distance (Kb) distribu-tion. White bars - keratinocytes expressing E6/E7; Black bars - keratinocytes expressing E6/E7 grown for 4 weeks in a folate-free medium.
