# Supplementary material for: Folate levels modulate oncogene-induced replication stress and tumorigenicity
Source: EMBO Mol Med. 2015 Jul 21;7(9):1138–52. doi: 10.15252/emmm.201404824 (PMC4568948; doi:10.15252/emmm.201404824)
Supplement: Supplementary file 2 [file emmm0007-1138-sd2.pdf]

EMBO
Molecular Medicine

Folate Levels Modulate Oncogene-Induced Replication Stress and Tumorigenicity

Noa Lamm, Karin Maoz, Assaf C. Bester, Michael M. Im, Donna S. Shewach, Rotem Karni and Batsheva Kerem

Corresponding author: Batsheva Kerem, The Hebrew University

Review timeline:

Submission date:	30 April 2013
Editorial Decision:	05 May 2013
Additional author correspondence :	22 October 2013
Additional Editorial Correspondence :	22 October 2013
Resubmission:	05 November 2014
Editorial Decision:	04 December 2014
Appeal:	07 December 2014
Editorial Decision:	19 December 2014
Revision received:	13 May 2015
Editorial Decision:	10 June 2015
Revision received:	26 June 2015
Accepted:	29 June 2015

Transaction Report:

(Note: With the exception of the correction of typographical or spelling errors that could be a source of ambiguity, letters and reports are not edited. The original formatting of letters and referee reports may not be reflected in this compilation.)

Editor: Céline Carret

1st Editorial Decision 05 May 2013

Thank you for the submission of your manuscript "Environmental Factors Modulate Oncogene-Induced Replication Stress and tumorigenicity".

I have now had the opportunity to carefully read your paper and the related literature and I have also discussed it with my colleagues. I am afraid that we concluded that the manuscript is not well suited for publication in EMBO Molecular Medicine and have therefore decided not to proceed with peer review.

The main reason for this decision is the unclear clinical significance of the findings at this stage. While we find the study interesting and conclusive in vitro, the patho-physiological relevance and implications of replication stress induction by folate deficiency and consequences on cancer development in an animal model in vivo remain uncertain. As such, I am afraid that the study does not fit our criteria for publication in EMBO Molecular Medicine.

I am sorry that I could not bring better news.

Additional author correspondence 22 October 2013

We have previously submitted to EMM a manuscript entitled ìEnvironmental Factors Modulate Oncogene-Induced Replication Stress and tumorigenicityî to be considered for publication in EMBO Molecular Medicine (EMM-2013-02975).

In this study we revealed that cellular DNA replication is affected by both environmental and genetic factors. We investigated the effect of folate, an environmental factor essential for nucleotide biosynthesis, on oncogene-induced cancer development. Our results show that folate deficiency, leads to replication stress resulting in DNA breaks in a concentration-dependent manner. Folate deficiency significantly enhances the oncogene-induced replication stress, leading to increased DNA damage and tumorigenicity in vitro. Importantly, we demonstrated that the extent of the replication stress is a key regulator of cancer development.

EMM decided not to send the manuscript for review based on: îThe main reason for this decision is the unclear clinical significance of the findings at this stage. While we find the study interesting and conclusive in vitro, the patho-physiological relevance and implications of replication stress induction by folate deficiency and consequences on cancer development in an animal model in vivo remain uncertain.ì

We have devoted a substantial amount of time and effort to meet this criticism. We have studied the effect of folate deficiency on cancer development in a mouse model. Our results show that oncogene-expressing cells grown under folate deficiency show significantly increased frequency of tumors in mice, underlining the essential role of an environmental factor in tumor initiation. Our results clearly demonstrate that folate deficiency significantly enhances tumor development caused by oncogene expression in vivo.

The abstract of the new manuscript version is attached.

In light of these new results we would like to ask that EMM will reconsider our manuscript for publication in EMM and allow us to submit the new version via the EMM website.

Additional Editorial Correspondence 22 October 2013

Thank you very much for your letter inquiring whether we would be interested in your improved article now including in vivo studies. After discussing within the team, I am happy to report that yes indeed, we would.

Please submit your article via the website at http://embomolmed.embopress.org.
Feel free to indicate that your new submission is a resubmission of article referenced EMM-2013-02975.

I am looking forward to seeing your article soon.

2nd Editorial Decision 04 December 2014

Thank you for the submission of your manuscript "Environmental Factors Modulate Oncogene-Induced Replication Stress and Tumorigenicity". We have now heard back from the three referees whom we asked to evaluate your manuscript.

As you will see, while one Reviewer is generally more positive, Reviewers 2 and 3 point to significant and important issues that, I am afraid, preclude publication of the manuscript in EMBO Molecular Medicine.

I will not discuss each point in detail but I would like to mention that in general, while the topic is of interest, the article suffers from limited conceptual advance and a correlative nature; together with the fact that we only accept papers that receive a majority of enthusiastic support upon initial review, I am afraid that these issues leave us no choice but to return the manuscript to you at this stage.

I am sorry to disappoint you on this occasion. I hope, however, that the Reviewers' comments will be helpful for your continued work in this area.

***** Reviewer's comments *****

Referee #1 (Comments on Novelty/Model System):

Chromosomal instability is a hallmark of cancer. Replication stress-induced DNA damage, caused by aberrant oncogene expression, plays a prominent role in driving genomic instability in early cancer stages, but how exactly replication is affected is still not clear. Even more, the effect of nutrients on replication stress is an unexplored field. Moreover, the work emphasizes the potential role of nutrients and diets in the prevention and treatment of cancer.
To address their questions and consolidate their findings the authors have used several epithelial cell lines and various oncogenis stimuli to show that the findings are global. Also they have used several assays to prove the tumorigenicity effect from their treatments.

Referee #1 (Remarks):

In the present report Lamm and collaborators address a very interesting question of how replication stress that is responsible for chromosomal instability is modulated from the earliest stages of cancer development. Specifically, they demonstrate that environmental and genetic factors can cooperate to modulate in a quantitative manner the replication stress that they exert. As an environmental factor they choose to regulate the supply of folate to normal cells, as a nutrient representative that regulates the intracellular nucleotide pool, and clearly depict that its deficiency leads to replication stress in a concentration-dependent manner. As genetic factors they use various oncogenes such as cyclin E, Ras and HPV16 E6/E7 oncogenes that are known replication stress inducers. By combining various degree of folate deficiency concomitantly or at different time phases with these oncogenes and in several cellular types the authors were able to demonstrate an enhancement of oncogene-induced replication stress, leading to increased DNA damage and tumorigenicity. An important result among the various interesting observations was the ability of cells that were allowed to recover a substantial time after folate deficiency to still be able to exhibit tumorigenicity.
Overall this is a very interesting work that provides significant advancement and shed light in elucidating the mechanistic(s) of the earliest stages of cancer development and specifically how replication stress can be affected in quantitative manner by combinations of environmental and genetic factors. Moreover, the work emphasizes the potential role of nutrients and diets in the prevention and treatment of cancer. I have no significant comments, apart from some very minor ones, as the work is also very well presented.

Minor points

1. In first section of Materials-Methods the authors describe only BJ cells and keratinocytes, while the other cell lines employed 3T3 and MCF-10A are mostly referred in other sections. I would suggest naming all cell lines used in the first section and if specific culture conditions are pertinent to a specific technique than the authors could simply state that this is described in the corresponding section.
2. Very few typos found: i) in Discussion page 14 line 18, the words "in-vivo" should be used as throughout the text (in vivo), and ii) second section of Materials-Methods the phrase "pBABE-puro based vector was a kindly provided by" should probably read "pBABE-puro based vector was kindly provided by".

Referee #2 (Comments on Novelty/Model System):

stastistical questions and wondering if experiments been repeated?

Referee #2 (Remarks):

This is a potentially interesting study by Lamm et al that seeks to address the combined effects of cellular and environmental factor in replication dynamics in cancer development. The paper suggest that lower folate levels decrease replication fork rate and increased active origins potentially due to a decreased nucleotide pool. They show that decreased folate levels in the presence of two different oncogenes increase colony formation and decrease tumor-free flanks in mice.

Overall the manuscript is very brief and preliminary. Also, as alterations in folate levels are known to decrease the nucleotide pool, the paper does not provide significant findings. Several figure panels lack error bars and it is not clear if these experiments have been repeated and therefore are reproducible. For example only two repeats performed for all fibre analysis? P- values surprisingly low. The replication stress induced by folate-depletion should be characterized in more detail (e.g. look at DDR, checkpoint activation, RPA and RAD51 foci formation). Methotrexate and 5-fluoracil are anti-folate drugs commonly used in cancer treatment, as the authors claim that decreased folate levels actually increase tumor development, the effects of oncogene induction together with antifolate drugs on colony formation, replication stress and tumor development in mice should be investigated.

Specific comments
1) Figure 1. The authors investigate how folate levels affect replication in normal immortalized cells (BJ TERT), in order to draw any conclusions if normal cells would show the same response to alterations in folate levels the authors should relate the folate concentrations used in the paper to physiological folate concentrations.

2) Figure 1 A: The authors state that the growth rate of BJ-hTERT cells is not affected when kept in normal or folate-free medium for 10 days. However, close inspection of the growth curves in Figure 1 A indicates that cells grown in folate-free media already exhibit a slowed down proliferation rate at this time point.

3) Figure 1A. It would help the readers if the authors could rearrange the data in the figure panels so that the reader easily can follow the logic in the sequence of experiments. Figure 1A is confusing for the reader as it is not fully explained in the Results section until after panel C and D, one possibility is to move out the data between day 14-48 in Figure 1A and put in a separate graph before figure 1E and 1F.

4) Figure 1 C: In the text the authors state a p-value of 1.6x10-32 for the DNA combing assay when comparing replication speed of cells grown in normal vs. folate-free medium. Given the variability of this assay such a low p-value is surprising. It is not clear from the text how the authors determined the p-value. Error bars are missing from this graph and should be included. It is also not clear how many independent repeats were performed for this experiment (from the text it sounds like it was only done twice with the first experiment shown in Figure 1C and the second in Figure E1). The number of analysed fibres (100) is also unusual low for these kind of experiments. As the difference between the fibre lengths is so dramatic it would be beneficial to show microscopic examples for both conditions.

5) Figure 1 D: Similar to Figure 1 C this experiment only has been performed twice, showing the corresponding results in Figure 1 D and E1.

6) Figure 1C and 1D.The authors rescue the decreased fork distance and replication rate upon decreased folate levels in Figure 1C and D by addition of nucleotides suggesting that an insufficient nucleotide pool is causing the observed replication stress upon folate deficiency. To further support this, it would be great if the authors could measure the nucleotide pool upon folate drop-out. As

folate deficiency causes uracil incorporation in DNA the authors should also measure the uracil incorporation into DNA.

7) Figure 1E and F. The authors should also investigate if they can rescue the phenotype/replication stress in Figure 1E and F by addition of nucleotides as in Figure 1C and D, as well as try if growth factor withdrawal causes a decrease in replication fork rate and distance in order to exclude that decreased proliferation generally causes replication stress and thus is a secondary effect upon folate deficiency.

8) Figure 2 A: Expression of the empty vector alone already reduces replication speed to 1.18 Kb/min when compared to 1.59 Kb/min in control cells from Figure 1.

9) Figure 2. The authors have previously shown that the cyclin E oncogene decreased the cellular nucleotide levels in the newly transformed cells, causing replication stress (Cell 2011). In Figure 1, they show that decreased folate levels might affect replication through decreased nucleotide levels. Thus it is not surprising that two treatments that cause replication stress together enhance the stress.

10) Figure 3A and B: The authors show that decreased folate levels increase colony numbers and in Figure 3E they show decreased tumor free flanks upon decreased folate levels. However in Figure 1A, they show that decreased folate levels decreases proliferation and that the cell cultures stop growing. Thus the data is not consistent between the figures, could the authors please explain the inconsistency between the experiments?

11) The title does not accurately reflect the focus of the paper. The title says "environmental factors", however only folate levels are investigated. Furthermore, folate is a dietary not an environmental factor.

Referee #3 (Comments on Novelty/Model System):

Results are not unexpected. However, it is interesting to readers because the manuscript emphasizes the significant of microenvironemnt in cancer development.

Referee #3 (Remarks):

In this study, the authors investigated the effect of low-concentration folate in culture medium on the cell's replication reaction and chromosomal instability. The readouts were replication speeds and sister fork distance measured by the fiber FISH technique, and gamma-H2AX staining. Results showed that the replication speed was declined, and the inter-fork distance was increased, suggesting that low-folate leads to replication stress. When cells incubated with low folate were tested for anchorage independent growth in semi-solid cultures and tumorigeniticy in nude mice, they found that cells that once had experienced a low folate condition showed increased frequency of transformation. Altogether, the authors suggest that folate deficiency, an environmental factor, cooperates synergistically with cancer-producing genetic mutations.
Experiments were generally well conducted. The conclusion, a synergy between environmental and genetic factors in cancer development, will be interesting to a broad range of readers of the Journal. I have one major concern, and several minor concerns.

Major

The authors clearly indicated the correlation between folate deficiency, replication stress, DNA double-strand breaks and tumorigenesis. However, it is not clear if replication stress is a cause of increased tumor formation. It is possible that DNA hypomethylation, which frequently accompanies folate deficiency, may have led activation of unidentified oncogenes, leading to the increased incidence of tumor formations. Numerous possibilities can be imagined as for the cause of enhanced transformation. Therefore, the authors should be cautious not describing that the replication stress is the cause of tumor progression in this system. For example, "that the extent of replication stress is a

key regulator of cancer development." In the last sentence of Abstract should be weakened.

Minor

1. Page 6, "(Figure 1A, compare cells grown in commercial DMEM to cells grown without folate at day 10),"
The data the authors referred to here does appear at day 7, instead of day 10.
2. Kb/min
Sometimes, "/" is wrongly printed (pages 9, 10).
3. Page 12, "Ras expression by itself significantly induced colony formation from 22 colonies per plate in the control cells"
"22" is likely to be "122".
4. Figure 3C is not mentioned in the main text.
5. Methods
"folate-free DMEM (custom-made, Biological Industries, Beit Haemek, Israel)"
"folate-free DMEM and normal DMEM (containing 9040nM folate)"
Need to describe how the folate concentration was actually measured as zero and 9040 nM, respectively. Or need to describe that the concentration is simply estimates.

Appeal 07 December 2014

Thank you very much for providing us with a thorough review of our manuscript. Most of the comments raised by the reviewers are highly important and can improve our main conclusions. However, we feel that rejection of our manuscript from publication in EMBO Molecular Medicine, based on the reviewers' comments may not be justified due to the overall positive and supportive review.

Reviewer 1: We find the review of reviewer 1, exquisitely supportive and enthusiastic. Among other superlatives this reviewer pointed out that "this is a very interesting work that provides significant advancement and shed light in elucidating the mechanistic(s) of the earliest stages of cancer development and specifically how replication stress can be affected in quantitative manner by combinations of environmental and genetic factors. Moreover, the work emphasizes the potential role of nutrients and diets in the prevention and treatment of cancer. I have no significant comments, apart from some very minor ones, as the work is also very well presented".

Reviewer 3: The review provided by reviewer 3 was also positive. This reviewer described the work as "very interesting to readers because the manuscript emphasizes the significance of microenvironment in cancer development". Moreover, the reviewer wrote "Experiments were generally well conducted. The conclusion, a synergy between environmental and genetic factors in cancer development, will be interesting to a broad range of readers of the Journal". The major concern of reviewer 3 was the role that replication stress caused by folate deficiency plays in tumor development: "It is possible that DNA hypomethylation, which frequently accompanies folate deficiency, may have led activation of unidentified oncogenes, leading to the increased incidence of tumor formations". Indeed, in the text of the manuscript it was not sufficiently explained/emphasized that the folate free medium used in the experiments, contains enough methyl sources to avoid hypomethylation due to folate deficiency. This will be now explained and emphasized.

Reviewer 2: This reviewer stated that the manuscript is a "potentially interesting study" however, raised several concerns. We are willing to perform the experimental and textual changes raised by this reviewer and are confident in our ability to address all the reviewer's major as well as minor concerns.

We would like to emphasize that our manuscript provides novel conceptual advances by showing that: 1.Replication stress can be affected in a quantitative manner by a combination of diet/nutritional and genetic factors to modulate cancer development. 2. Cells that were allowed to recover a substantial time after folate deficiency still able to exhibit tumorigenicity, both in vitro and

in vivo.

In light of the above, we are baffled by the decision to reject our manuscript and would like to kindly ask that this decision would be reconsidered. We are more than willing to address all of the reviewer's remarks and to submit a revised version of the manuscript.

3rd Editorial Decision 19 December 2014

I apologize for the delay in getting back to you. Just before Christmas, we always have a surge of submissions and therefore, appeals get even more delayed.

I have now read attentively your letter and discussed with my colleague. In light of your arguments, we would like to give you a chance to address all referees' comments in full. I would also add that the revised manuscript will be re-vealuated by the same reviewers and I cannot guarantee that they will be satisfied to the point that they will support publication.

Please note that it is EMBO Molecular Medicine policy to allow only a single round of revision and that, as acceptance or rejection of the manuscript will depend on another round of review, your responses should be as complete as possible.

EMBO Molecular Medicine has a "scooping protection" policy, whereby similar findings that are published by others during review or revision are not a criterion for rejection. Should you decide to submit a revised version, I do ask that you get in touch after three months if you have not completed it, to update us on the status.

Please also contact us as soon as possible if similar work is published elsewhere. If other work is published we may not be able to extend the revision period beyond three months.

I look forward to receiving your revised manuscript.

1st Revision - authors' response 13 May 2015

Attached please find our revised manuscript entitled "**Folate Levels Modulate Oncogene-Induced Replication Stress and Tumorigenicity**" in which we have addressed **all the points raised by the reviewers,** including the requested additional experiments and text changes.

Our responses are detailed below point-by-point.

Please note that as requested by reviewer 2, the title of the manuscript was slightly changed ("environmental factors" was replaced by "Folate levels"). In addition, as requested by reviewer 2, we have analyzed the nucleotide intracellular levels and therefore added additional authors (Im and Shewach), who performed the analysis.

We hope that the paper will now be found suitable for publication in *EMBO Molecular Medicine*.

Referee #1 (Comments on Novelty/Model System):

Chromosomal instability is a hallmark of cancer. Replication stress-induced DNA damage, caused by aberrant oncogene expression, plays a prominent role in driving genomic instability in early cancer stages, but how exactly replication is affected is still not clear. Even more, the effect of nutrients on replication stress is an unexplored field. Moreover, the work emphasizes the potential role of nutrients and diets in the prevention and treatment of cancer.
To address their questions and consolidate their findings the authors have used several epithelial cell lines and various oncogenis stimuli to show that the findings are global. Also they have used several assays to prove the tumorigenicity effect from their treatments.

There were no comments to address

Referee #1 (Remarks):

In the present report Lamm and collaborators address a very interesting question of how replication stress that is responsible for chromosomal instability is modulated from the earliest stages of cancer development. Specifically, they demonstrate that environmental and genetic factors can cooperate to modulate in a quantitative manner the replication stress that they exert. As an environmental factor they choose to regulate the supply of folate to normal cells, as a nutrient representative that regulates the intracellular nucleotide pool, and clearly depict that its deficiency leads to replication stress in a concentration-dependent manner. As genetic factors they use various oncogenes such as cyclin E, Ras and HPV16 E6/E7 oncogenes that are known replication stress inducers. By combining various degree of folate deficiency concomitantly or at different time phases with these oncogenes and in several cellular types the authors were able to demonstrate an enhancement of oncogene-induced replication stress, leading to increased DNA damage and tumorigenicity. An important result among the various interesting observations was the ability of cells that were allowed to recover a substantial time after folate deficiency to still be able to exhibit tumorigenicity.
Overall this is a very interesting work that provides significant advancement and shed light in elucidating the mechanistic(s) of the earliest stages of cancer development and specifically how replication stress can be affected in quantitative manner by combinations of environmental and genetic factors. Moreover, the work emphasizes the potential role of nutrients and diets in the prevention and treatment of cancer. I have no significant comments, apart from some very minor ones, as the work is also very well presented.

There were no comments to address – the reviewer was highly enthusiastic about our work.

Minor points

1. In first section of Materials-Methods the authors describe only BJ cells and keratinocytes, while the other cell lines employed 3T3 and MCF-10A are mostly referred in other sections. I would suggest naming all cell lines used in the first section and if specific culture conditions are pertinent to a specific technique than the authors could simply state that this is described in the corresponding section.

As requested by the reviewer, we added a description of the missing cell lines (3T3 and MCF10A) and details of their specific culture conditions to the paragraph entitled "Cell cultures". Please see page 21 lines 13-21.

2. Very few typos found: i) in Discussion page 14 line 18, the words "in-vivo" should be used as throughout the text (in vivo), and ii) second section of Materials-Methods the phrase "pBABE-puro based vector was a kindly provided by" should probably read "pBABE-puro based vector was kindly provided by".

The typos were corrected. Please see pages 17 and 22 lines 18 and 4, respectively.

Referee #2 (Comments on Novelty/Model System):

stastistical questions and wondering if experiments been repeated?

As requested by the reviewer we repeated the experiments where needed so that all experiments have now been repeated at least three times. For each experiment the number of repeats is mentioned in the text and in the figure legends. We also addressed the comments on the statistics, as described below in response to the specific comments.

Referee #2 (Remarks):

This is a potentially interesting study by Lamm et al that seeks to address the combined effects of cellular and environmental factor in replication dynamics in cancer development. The paper suggest that lower folate levels decrease replication fork rate and increased active origins potentially due to a decreased nucleotide pool. They show that decreased folate levels in the presence of two different oncogenes increase colony formation and decrease tumor-free flanks in mice.

Overall the manuscript is very brief and preliminary. Also, as alterations in folate levels are known to decrease the nucleotide pool, the paper does not provide significant findings. Several figure panels lack error bars and it is not clear if these experiments have been repeated and therefore are reproducible. For example only two repeats performed for all fibre analysis? P- values surprisingly low. The replication stress induced by folate-depletion should be characterized in more detail (e.g. look at DDR, checkpoint activation, RPA and RAD51 foci formation). Methotrexate and 5-fluoracil are anti-folate drugs commonly used in cancer treatment, as the authors claim that decreased folate levels actually increase tumor development, the effects of oncogene induction together with antifolate drugs on colony formation, replication stress and tumor development in mice should be investigated.

We have divided the reviewers' comments in order to respond to each and every comment:

Overall the manuscript is very brief and preliminary.

We have performed the additional experiments required by the reviewer and thus believe that the revised version is no longer brief and preliminary.

Also, as alterations in folate levels are known to decrease the nucleotide pool, the paper does not provide significant findings.

Indeed, folate levels are known to decrease the nucleotide pool. However, folate deficiency is generally thought to affect DNA stability primarily through uracil misincorporation during DNA synthesis, which leads to catastrophic DNA repair cycles (reviewed in (Blount & Ames, 1995; Duthie, 2011; Fenech, 2012). Our findings however, shed light on an earlier effect of folate deficiency on genome stability, as we showed that folate deficiency perturbed the replication dynamics and resulted in replication stress-induced genome instability. In the revised version we have emphasized this point (please see page 18 lines 1-2).

Furthermore, one of the significant novel findings in our study is that replication stress can be affected in a quantitative manner, by combinations of dietary and genetic factors. An additional significant finding is that cells that were allowed to recover a substantial time after folate deficiency still promoted tumorigenicity.

Several figure panels lack error bars and it is not clear if these experiments have been repeated and therefore are reproducible. For example only two repeats performed for all fibre analysis? P- values surprisingly low.

As mentioned above, we repeated the replication dynamic experiments and now all experiments have at least three repeats. The distributions of replication rate and fork distance do not show error bars because they represent the results of one experiment. The results from additional experiments can be found in the supplementary information (Figures E1-4). As requested by the reviewer, we have added the average of three experiments for each condition (n>350) (please see new Figure 1E and G and Figure 2E and G) in the form of box-plots.

The P- values were calculated using two-tailed T test, which is appropriate for the analysis of normal distributions of replication rate and fork distance data (please see "statistical analysis" in Conti *et al*, Mol Biol Cell, 2007). We would like to mention that similarly low p-values have also been found in other DNA combing analyses (please see Figure 4 in Gay *et al*, EMBO reports, 2010).

The replication stress induced by folate-depletion should be characterized in more detail (e.g. look at DDR, checkpoint activation, RPA and RAD51 foci formation).

As the reviewer suggested we have now expanded our study and investigated the effect of folate deficiency on additional DDR and signal transduction pathways. This includes studying the level of phosphorylated-ATM, phosphorylated-CHK1 and RAD51 foci formation (please see new Figure 4 and the related text on page 13 and 14). As can be seen in the figure, the combination of folate deficiency and oncogene expression resulted in enhanced activation of the DDR.

Methotrexate and 5-fluoracil are anti-folate drugs commonly used in cancer treatment, as the authors claim that decreased folate levels actually increase tumor development, the effects of oncogene induction together with antifolate drugs on colony formation, replication stress and tumor development in mice should be investigated.

We thank the reviewer for this comment regarding the implications of anti-folate therapy. In fact, anti-folate drugs and a folate deficient medium have the same effect on the nucleotide pool (Shane, 1989). However, these conditions have a dual effect on the tumorigenic potential of the cells, depending on the duration and extent of folate deficiency or antifolate drugs and on the cell stage (tumorigenicity). The following paragraph was added to the Introduction of the revised manuscript (please see Introduction page 5 lines 5-21):

"Folate deficiency has a dual effect on the tumorigenic potential of the cells depending on the duration and extent of the folate deficiency and on the cell stage (tumorigenicity). In neoplastic cells there is extensive DNA replication and cell division. In these cells folate deficiency causes ineffective DNA synthesis, resulting in inhibition of tumor growth (Kim, 1999; Choi & Mason, 2002). Indeed, this has been the basis for cancer chemotherapy using a number of antifolate agents (for example, methotrexate and 5-fluorouracil) (Kim, 1999; Choi & Mason, 2002). Like most chemotherapies, anti-folate drugs are toxic to both normal and neoplastic cells and prolonged folate deficiency eventually results in growth arrest and cell death regardless of the tumorigenicity of the cells. However, under shorter and milder folate deficiency conditions neoplastic cells and other extensive proliferating cells will die whereas normal cells will survive. An accumulating body of epidemiological, clinical, and experimental evidence suggests that normal cells that survived folate deficiency are predisposed to neoplastic transformation (Kim, 1999; Choi & Mason, 2002). This dual effect of folate deficiency, which is also known as the "double-edged sword" effect, explains why methotrexate therapy is associated with increased risk of secondary malignancy (Schmiegelow *et al*, 2009)."

This point is also discussed in the Discussion part (please see page 19 lines 3-17).

Specific comments

1) Figure 1. The authors investigate how folate levels affect replication in normal immortalized cells (BJ TERT), in order to draw any conclusions if normal cells would show the same response to alterations in folate levels the authors should relate the folate concentrations used in the paper to physiological folate concentrations.

As the reviewer suggested it would be valuable to relate the *in vitro* values to physiological values. This is extremely challenging, primarily because folate is supplemented in tissue culture media as folic acid while *in vivo* it is provided through nutrition in the form of various folate derivatives, whose cellular uptake is much more efficient than the uptake efficiency of folic acid. Moreover, differences among individuals in the efficiency to absorb and metabolize this vitamin (reviewed in (Fenech, 2012) also affect the actual folate level *in vivo*. Further epidemiological, clinical and interventional studies are required to determine the physiological levels of folate deficiency and the deficiency duration that affect replication dynamics.

This paragraph was added to the Discussion (please see page 18 lines 19-25).

2) Figure 1 A: The authors state that the growth rate of BJ-hTERT cells is not affected when kept in normal or folate-free medium for 10 days. However, close inspection of the growth curves in Figure 1A indicates that cells grown in folate-free media already exhibit a slowed down proliferation rate at this time point.

This comments is right, of course. We apologize for the inconsistency in our writing. The experiments were performed in cells grown in a folate deficient medium for 7 days and not for 10 days. In some sections it was mistakenly written 10 days. This is an unfortunate mistake that has now been corrected.

3) Figure 1A. It would help the readers if the authors could rearrange the data in the figure panels so that the reader easily can follow the logic in the sequence of experiments. Figure 1A is confusing for the reader as it is not fully explained in the Results section until after panel C and D, one possibility is to move out the data between day 14-48 in Figure 1A and put in a separate graph before figure 1E and 1F.

As requested by the reviewer we rearranged the data in Figure 1A. We removed some of the data from Figure 1A and added it to Figure 2A. Now the reader easily can follow the logic behind the sequence of experiments.

4) Figure 1C: In the text the authors state a p-value of 1.6x10-32 for the DNA combing assay when comparing replication speed of cells grown in normal vs. folate-free medium. Given the variability of this assay such a low p-value is surprising. It is not clear from the text how the authors determined the p-value.

As mentioned above (please see page 4 lines 17-21 in this letter) the P- values were calculated using two-tailed T test, which are appropriate for the analysis of normal distributions of replication rate and fork distance data (please see "statistical analysis" in Conti *et al*, Mol Biol Cell, 2007). We would like to mention that similarly low p-values have also been found in other DNA combing analyses (for example in (please see Figure 4 in Gay *et al*, EMBO reports, 2010).

Error bars are missing from this graph and should be included. It is also not clear how many independent repeats were performed for this experiment (from the text it sounds like it was only

done twice with the first experiment shown in Figure 1C and the second in Figure E1). The number of analysed fibres (100) is also unusual low for these kind of experiments.

As mentioned above (please see page 4 line 8-15 in this letter), we repeated the replication dynamic experiments and now all experiments have at least three repeats. The distributions of replication rate and fork distance do not show error bars because they represent the results of one experiment. Results from the additional experiments can be found in the supplementary information (Figures E1-4). As requested by the reviewer, we have added the average of the three experiments for each condition (n>350) (please see new Figures 1E and G and Figures 2E and G) in the form of box-plots.

As the difference between the fibre lengths is so dramatic it would be beneficial to show microscopic examples for both conditions.

As suggested, we have added microscopic examples for both conditions. Please see new Figure 1C.

5) Figure 1D: Similar to Figure 1C this experiment only has been performed twice, showing the corresponding results in Figure 1D and E1.

As explained in our response to point 4, we repeated the replication dynamic experiments so now we have three repeats summarized in the form of a box plot (please see new Figures 1E and G and Figures 2E and G).

6) Figure 1C and 1D.The authors rescue the decreased fork distance and replication rate upon decreased folate levels in Figure 1C and D by addition of nucleotides suggesting that an insufficient nucleotide pool is causing the observed replication stress upon folate deficiency. To further support this, it would be great if the authors could measure the nucleotide pool upon folate drop-out. As folate deficiency causes uracil incorporation in DNA the authors should also measure the uracil incorporation into DNA.

As requested by the reviewer, using the high-performance liquid chromatography (HPLC) method, we measured the concentration of dNTPs with and without folate. As expected, the cellular dTTP level in cells grown under a folate deficiency for 3-30 days was significantly lower than the level in the same cells grown in a normal medium. The levels of the dATP, dGTP and dCTP were below detection. Please see the new supplementary figure E2.

7) Figure 1E and F. The authors should also investigate if they can rescue the phenotype/replication stress in Figure 1E and F by addition of nucleotides as in Figure 1C and D, as well as try if growth factor withdrawal causes a decrease in replication fork rate and distance in order to exclude that decreased proliferation generally causes replication stress and thus is a secondary effect upon folate deficiency.

As requested by the reviewer we conducted a rescue experiment (repeated three times) in which cells were grown in 100nM folate for 7 days and were rescued by nucleoside supplementation (Figure 2 D-G).

As can be seen in the proliferation curve (Figure 2A), cells grown under 100nM folate proliferated normally until day 21. This indicates that the replication stress is not the result of impaired proliferation.

8) Figure 2A: Expression of the empty vector alone already reduces replication speed to 1.18 Kb/min when compared to 1.59 Kb/min in control cells from Figure 1.

Replication dynamics can vary between experiments, since they are affected by several factors including the confluency of the cells, their passage, and time after oncogene expression, etc. Therefore, we always compare the average rate and fork distance between conditions in a single experiment. Nevertheless, in the box plots it can be seen that though the average replication rate or fork distance for a specific condition can vary between different experiments, it does not mask the effect of folate deficiency or oncogene expression on the replication dynamics.

9) Figure 2. The authors have previously shown that the cyclin E oncogene decreased the cellular nucleotide levels in the newly transformed cells, causing replication stress (Cell 2011). In Figure 1, they show that decreased folate levels might affect replication through decreased nucleotide levels. Thus it is not surprising that two treatments that cause replication stress together enhance the stress.

Though not surprising, the results demonstrate for the first time that enhanced replication stress can enhance tumorigenicity.

10) Figure 3A and B: The authors show that decreased folate levels increase colony numbers and in Figure 3E they show decreased tumour free flanks upon decreased folate levels. However in Figure 1A, they show that decreased folate levels decreases proliferation and that the cell cultures stop growing. Thus the data is not consistent between the figures, could the authors please explain the inconsistency between the experiments?

We thank the reviewer for the request to better explain the differences between the results in the two figures.

The protocol of the colony formation and tumour development in mice (old figures 3A and B) was different from the protocol used for the proliferation assay (Figure 1A). **The proliferation curves** were analysed in cells grown under folate deficient conditions, at various time points (as indicated in the figure). Whereas, **the soft agar and the tumorigenesis assays in nude mice** were performed in cells grown for 4 weeks in a mild folate-deficient medium (100nM) and then for 2 more weeks in a normal medium (Figure 5A and B), to allow for recovery of the cells from proliferation arrest due to the prolonged growth in folate-deficient conditions. This enabled us to evaluate the tumorigenicity potential of cells due to folate deficiency-induced DNA damage.

We would like to emphasize that the results from the "recovery" experiments imply that even a transient folate deficiency is sufficient to disrupt genome integrity and enhance tumorigenicity, as DNA damage that had been generated under conditions of folate deficiency is irreversible and thus cannot be recovered by later folate supplementation.

We have now added this missing rationale of the protocol to the text (please see Results section page 15 line 7-12 and in the Discussion page 19 lines 3-17 and the legend to Figure 5).

11) The title does not accurately reflect the focus of the paper. The title says "environmental factors", however only folate levels are investigated. Furthermore, folate is a dietary not an environmental factor.

As suggested, the title has been changed. The new title is "Folate Levels Modulate Oncogene-Induced Replication Stress and Tumorigenicity".

We also have replaced the term "environmental factors" throughout the text by "dietary" or "non-genetic".

Referee #3 (Comments on Novelty/Model System):

Results are not unexpected. However, it is interesting to readers because the manuscript emphasizes the significant of microenvironment in cancer development.

Referee #3 (Remarks):

In this study, the authors investigated the effect of low-concentration folate in culture medium on the cell's replication reaction and chromosomal instability. The readouts were replication speeds and sister fork distance measured by the fiber FISH technique, and gamma-H2AX staining. Results showed that the replication speed was declined, and the inter-fork distance was increased, suggesting that low-folate leads to replication stress. When cells incubated with low folate were tested for anchorage independent growth in semi-solid cultures and tumorigeniticy in nude mice, they found that cells that once had experienced a low folate condition showed increased frequency of transformation. Altogether, the authors suggest that folate deficiency, an environmental factor, cooperates synergistically with cancer-producing genetic mutations.
Experiments were generally well conducted. The conclusion, a synergy between environmental and genetic factors in cancer development, will be interesting to a broad range of readers of the Journal. I have one major concern, and several minor concerns.

Major
The authors clearly indicated the correlation between folate deficiency, replication stress, DNA double-strand breaks and tumorigenesis. However, it is not clear if replication stress is a cause of increased tumour formation. It is possible that DNA hypomethylation, which frequently accompanies folate deficiency, may have led activation of unidentified oncogenes, leading to the increased incidence of tumour formations. Numerous possibilities can be imagined as for the cause of enhanced transformation. Therefore, the authors should be cautious not describing that the replication stress is the cause of tumour progression in this system. For example, "that the extent of replication stress is a key regulator of cancer development." In the last sentence of Abstract should be weakened.

We accept this comment and tuned down the role of replication stress in cancer development due to folate deficiency. Changes were made in the Abstract, Introduction (page 6 lines 3-5) and Discussion (Page 20 lines 1-2).

Minor
1. Page 6, "(Figure 1A, compare cells grown in commercial DMEM to cells grown without folate at day 10),"
The data the authors referred to here does appear at day 7, instead of day 10.

This comments is right, of course. We apologize for the inconsistency in our writing. The experiments were performed in cells grown in a folate deficient medium for 7 days and not for 10 days. In some sections it was mistakenly written 10 days. This is an unfortunate mistake that has now been corrected.

2. Kb/min
Sometimes, "/" is wrongly printed (pages 9, 10).

Corrected.

3. Page 12, "Ras expression by itself significantly induced colony formation from 22 colonies per plate in the control cells"
"22" is likely to be "122".

We are afraid that this is simply a misunderstanding. The number of colonies in the **control cells grown** in a normal medium is 22. The number of colonies following RAS expression in cells grown in a normal medium is 134.

4. Figure 3C is not mentioned in the main text.

Corrected.

5. Methods
"folate-free DMEM (custom-made, Biological Industries, Beit Haemek, Israel)"
"folate-free DMEM and normal DMEM (containing 9040nM folate)"
Need to describe how the folate concentration was actually measured as zero and 9040 nM, respectively. Or need to describe that the concentration is simply estimate.

We added that the folate concentrations were estimated by the manufacturer (please see Materials and Methods section page 21 line 6).

4th Editorial Decision 10 June 2015

Thank you for the submission of your revised manuscript to EMBO Molecular Medicine. We have now received the enclosed report from the most critical referee in the 1st round.

You will see from the comments pasted below that referee 2 is not fully satisfied by the revision and is requesting further experiments and details. While we do agree with referee 2 that the specific comments point 1) should be experimentally addressed to strengthen the new data by providing important controls, general point 1) and the specific comments points 3) and 4) should be addressed in writing in the main text. As the two points 2) were critical for this referee who requested additional experimental work and in order to make a better informed decision, I have asked the initial referee 1 to cross-comment on these and s/he replied the following:
Regarding the general point 2), this referee found that given existing literature (including the one you cited in your rebuttal letter), the approach you have taken is sufficient but a better and clearer bibliographic explanation is needed and should be included also in the main text.
Regarding specific comment 2), as you assessed intracellular levels of dNTPs pool already and given the very low levels of dNTPs, it is expected that uracil incorporation into DNA should take place in this condition and this should be commented upon in the main text with support from appropriate literature.

Please submit your revised manuscript as soon as possible.

***** Reviewer's comments *****

Referee #2 (Comments on Novelty/Model System):

fine system

Referee #2 (Remarks):

The authors have addressed many concerns raised by me and the other reviewers, which has improved the paper. However, many questions still need to be addressed.

1) The authors claim that they have repeated all experiments three times at least and that the number of repeats is mentioned in the text and figure legend. However, this is still lacking in a few experiments such as Figure 1A, 2A, 3E, 4A, 4D and it is therefore not clear if these experiments have been repeated at all.
2) The authors have not investigated the effects of oncogene induction together with antifolate drugs on colony formation, replication stress and tumor development in mice as suggested by the reviewer. They refer to a reference from 1989 that anti-folate drugs and folate deficient medium have same effect on the nucleotide pool however, this does not answer the comment.

Specific comments:

1) Figure 4A. The authors should investigate total ATM and CHK1 levels to exclude the possibility that the changed phosphorylation levels of ATM and CHK1 upon CycE and Folate treatment is not due to a reduction in total ATM and CHK1 protein levels.
2) The authors have not measured the uracil incorporation into DNA as suggested by the reviewer. As the authors claim that folate deficiency is known to affect DNA stability mainly through uracil incorporation it would be important to look at the uracil incorporation at the time for decreased DNA replication speed to clarify which is the main mechanism for the decreased cell growth.
3) The authors response that there "results demonstrate for the frista tima that enhanced replication stress can enhance tumorigenicity " are fundamentally wrong. Replication stress is well known to increase tumorigenicity, see Aguilera et al. 2015 Nature (Replication stress and cancer).
4) As different folate levels were used between the figures, sometime -folate means no folate (Figure 1) and sometimes 100nM (Figure 5), it would be better to not write "-folate" when folate is present but to write the concentration is order to make it easier for the reader to interpret the data.

2nd Revision - authors' response 26 June 2015

We are now resubmitting our manuscript entitled: "Folate Levels Modulate Oncogene-Induced Replication Stress and Tumorigenicity". We have addressed all the points that you kindly raised in your letter to us, which include the control experiment and text additions to address the comments by reviewer #2 and other comments.

Please see our point-by-point response below.

Thanking you for your assistance in finalizing the acceptance of the manuscript for publication in EMM.

Response to the comments of referee #2 (Remarks):

The authors have addressed many concerns raised by me and the other reviewers, which has improved the paper. However, many questions still need to be addressed.

1) The authors claim that they have repeated all experiments three times at least and that the number of repeats is mentioned in the text and figure legend. However, this is still lacking in a few experiments such as Figure 1A, 2A, 3E, 4A, 4D and it is therefore not clear if these experiments have been repeated at all.

We have now generated a table (supplementary table) which summarizes all the data regarding the number of repeats, the exact sample size (n), p-values, for each experiment (please see Table E1). As can be seen in the Table, the experiments presented in Figure 1A were repeated 3 times; 2A – 3 times; 3E – 3 times; 4A – 3 times; 4D – twice.

2) The authors have not investigated the effects of oncogene induction together with antifolate drugs on colony formation, replication stress and tumor development in mice as suggested by the reviewer. They refer to a reference from 1989 that anti-folate drugs and folate deficient medium have same effect on the nucleotide pool however, this does not answer the comment.

As suggested by the editor and reviewer 1 we added to the revised manuscript a better and clearer bibliographic explanation of why using folate deficiency is relevant and comparable to the use of antifolate drugs and even has several technical advantages (please see page 20 lines 13-25).

Specific comments:

1) Figure 4A. The authors should investigate total ATM and CHK1 levels to exclude the possibility that the changed phosphorylation levels of ATM and CHK1 upon CycE and Folate treatment is not due to a reduction in total ATM and CHK1 protein levels.

As requested by the reviewer we have performed Western blots controls, analyzing the level of ATM and CHK1 (non-phosphorylated). The results showed no changes in the level of the proteins, hence supporting our conclusion that changes in phosphorylated ATM and CHK1 are the result of replication stress and DNA damage. The results are presents in a new panel added to Figure 4A.

2) The authors have not measured the uracil incorporation into DNA as suggested by the reviewer. As the authors claim that folate deficiency is known to affect DNA stability mainly through uracil incorporation it would be important to look at the uracil incorporation at the time for decreased DNA replication speed to clarify which is the main mechanism for the decreased cell growth.

As suggested by the Editor and Reviewer 1, we have added to the manuscript a comment (supported by the literature) that the levels of the dTTP under folate deficiency were very low, hence uracil incorporation is expected (please see Page 8 lines 19-21).

3) The authors response that there "results demonstrate for the frista tima that enhanced replication stress can enhance tumorigenicity " are fundamentally wrong. Replication stress is well known to increase tumorigenicity, see Aguilera et al. 2015 Nature (Replication stress and cancer).

This is probably a misunderstanding of our explanation to the reviewer's comment no 9 in the previous point by point response.

We did not mean that our results show for the first time that replication stress leads to tumorigenicity. Our results show for the first time that replication stress is a **quantitative trait** and its extent has an enhanced effect on tumorigenicity. We thoroughly went over the conclusion in the manuscript and think that this point is clearly explained (please see for example page 21 lines 8-10).

4) As different folate levels were used between the figures, sometime -folate means no folate (Figure 1) and sometimes 100nM (Figure 5), it would be better to not write "-folate" when folate is present but to write the concentration is order to make it easier for the reader to interpret the data.

As suggested by the reviewer we have now corrected it in the text and figures so experiments of no folate were described as –folate, and when low concentrations of folate were added, the exact concentration is mentioned.
